# Vector semantics of multidomain protein architectures

**DOI:** 10.1093/bioadv/vbag037

**Published:** 2026-03-02

**Authors:** Xiaoyue Cui, Yuting Xiao, Maureen Stolzer, Dannie Durand

**Affiliations:** Ray and Stephanie Lane Computational Biology Department, Carnegie Mellon University, Pittsburgh, PA 15213, United States; Department of Biological Sciences, Carnegie Mellon University, Pittsburgh, PA 15213, United States; Department of Biological Sciences, Carnegie Mellon University, Pittsburgh, PA 15213, United States; Ray and Stephanie Lane Computational Biology Department, Carnegie Mellon University, Pittsburgh, PA 15213, United States; Department of Biological Sciences, Carnegie Mellon University, Pittsburgh, PA 15213, United States

## Abstract

**Summary:**

Multidomain proteins are mosaics of *domains*, protein modules that are associated with a specific structure or function and are found in diverse combinations. This modular organization facilitates the evolution of novel protein functions, but the principles that govern the relationship between the domain content of a protein and its function is poorly understood. In particular, do domains always contribute the same function, or does the functional contribution of a domain depend on the neighboring domains in the protein? To answer this question, we used vector embeddings, which account for local contextual signals, to model the protein domain content of multidomain proteins. We observe that multidomain architectures that are semantically similar share more functional attributes than multidomain architectures selected based on domain content similarity, alone, suggesting that context is important for understanding the relationship between domain content and protein function. Surprisingly, vector semantics also identified multidomain architecture pairs with significantly high functional similarity, despite having no domains in common at all, suggesting that vector semantics may be discovering domain “synonyms”. Taken together, our results underscore the importance of contextual models for understanding the interplay between domain architecture evolution and functional innovation in multidomain proteins.

**Availability and implementation:**

The data and code used in this work are available at https://zenodo.org/records/15769961.

## Introduction

**Figure 1:**
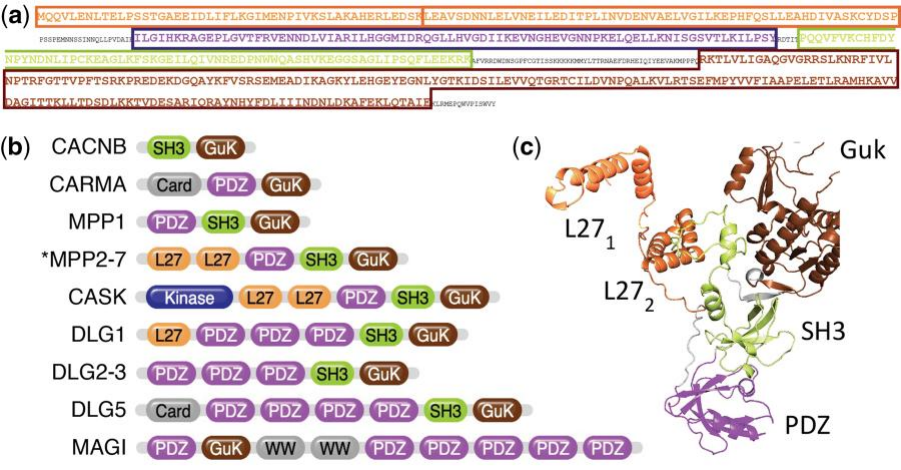
Example of a multidomain protein family: the Membrane-Associated Guanylate Kinases (MAGuKs). (a) Domains in sequence are identified by comparison with probabilistic models (e.g. HMMs) in a domain architecture database. (b) Domain architectures of MAGuK subfamilies exhibit similar domain content in various combinations. (c) Structure of MPP6, with individual domains color coded as in (b).

Multidomain proteins are mosaics of structural or functional units called domains. Domains act as protein modules, are found in otherwise unrelated proteins, and fold independently in many different contexts. The *domain architecture* (DA) of a protein is an abstract representation consisting of its domains in N- to C-terminal order. For example, the domain architecture of the Mpp6 protein shown in Fig. 1 can be represented as L27-L27-PDZ-SH3-GuK.

The domain architecture abstraction is widely used to probe questions of protein evolution, including variation in the domain repertoire across taxonomic lineages (Karev *et al.* 2004, Ye and Godzik 2004, Tordai *et al.* 2005, Cromar *et al.* 2016, Dohmen *et al.* 2020), plasticity in domain order (Bashton and Chothia 2002, Weiner 3rd *et al.* 2006, Kummerfeld and Teichmann 2009), domain occurrence graphs (Karev *et al.* 2002, Vogel *et al.* 2005, Przytycka *et al.* 2006, Cromar *et al.* 2014), and domain promiscuity, i.e. the propensity of a domain to co-occur with many other domains (Marcotte *et al.* 1999; Weiner *et al.* 2008, Basu *et al.* 2009, 2008, Cohen-Gihon *et al.* 2011, Cromar *et al.* 2014).

Multidomain sequences evolve via the insertion, internal duplication, and deletion of domains, resulting in families with similar domain architectures, but with some variation in domain content, order and copy number (e.g. the Membrane Associated Guanylate Kinases (MAGuKs) (Te Velthuis *et al.* 2007) shown in Fig. 1).

The modular organization of multidomain proteins supports rapid evolutionary exploration of new protein functions through the formation of different domain combinations that enable diverse biological roles. At the same time, this exploration is not unconstrained: the number of domain combinations observed in the multidomain repertoire is much smaller than expected by chance (Vogel *et al.* 2005, Yu *et al.* 2019, Cui *et al.* 2022). The principles that govern which domain combinations are realized in nature are poorly understood. Many forces could be contributing to those constraints, including structural compatibility, mutational processes, and cellular environment.

Here we focus on the relationship between the domain content of a protein and its function. In specific cases, the function of multidomain proteins can be understood in terms of the functions of individual domains. For example, the extracellular domains of a receptor kinase recognize a molecular signal; the intercellular kinase domain passes on that signal by phosphorylating an amino acid. More generally, proteins with identical domain architectures have more similar GO annotations, on average, than those that do not and proteins that share at least one domain have greater GO similarity than those that have no domains in common (Radivojac 2022). This is further supported by recent successes with protein function prediction methods that incorporate domain content information (e.g. You *et al.* 2018, Ibtehaz *et al.* 2023, Chen and Luo 2024, Hallee *et al.* 2024, Hu and Zhao 2025, Talo and Bozdag 2025, Wang *et al.* 2025). However, it is not clear whether a given domain confers the same functional properties in all contexts, or whether the functional contribution of a domain varies depending on neighboring domains in the protein.

### Vector semantics

Here we investigate *vector semantic* models, developed for natural language processing (NLP), as an analytical framework for probing the relationship between the domain content and the function of a multidomain protein. Just as documents are strings of words, domain architectures are strings of domains, suggesting that NLP models are a promising approach for exploratory analysis of the multidomain universe. Vector semantics are based on the assumption that the meanings of words are implicitly encoded in their usage in the language. The distribution of words across documents is a representation of word usage; hence, words that occur in similar contexts tend to have related meanings. Contextual information can be encoded as a *word embedding*, where each word corresponds to a vector that represents its distribution in a corpus of documents. The goal is to select a vectorization such that words that are proximal in the vector space will tend to have related meanings. This includes words with similar meanings (e.g. chilly, cool), but also words that have meanings that are dissimilar, but are topically related (e.g. *bus, train*; *dog, animal*; or *cup, coffee*).

Embeddings used in information retrieval are sparse, high dimensional encodings. In a term frequency—inverse document frequency (TF-IDF) encoding, each dimension corresponds to a document; the $j^{th}$ component of a word vector represents its frequency in document *j*, normalized by its frequency across the corpus (Luhn 1957, Sparck Jones 1972). In a TF-IDF vectorization, words that occur in the same documents will be close in the vector space. In a Pointwise Mutual Information (PMI) (Church and Hanks 1990) embedding, each dimension corresponds to a word in the vocabulary; the $j^{th}$ component of a word vector is a function of its frequency of co-occurrence with the $j^{th}$ word in the vocabulary, relative to the independent overall frequencies of the two words. In a PMI embedding, words will be proximal if they co-occur with the same words. Static language embeddings, e.g. Word2Vec (Mikolov *et al.* 2013), learn compact vector representations from training data (e.g. document corpora) by training a shallow neural network to predict nearby domains (context) given a target domain, with the skip-gram model. The learned embeddings reflect patterns of local adjacency within domain architectures. The dimensionality of the embedding is a user-defined parameter *m*. The context of a domain $D_{i}$ is defined to be the *w* domains immediately preceding and the *w* domains immediately following $D_{i}$ in the architecture. If fewer than *w* domains precede (or follow) $D_{i}$, then the flanking window(s) are truncated. Thus, the context includes up to 2*w* surrounding domains. Under this model, domains that tend to be flanked by similar sets of domains will be close in the embedding space.

Given a word embedding, a *document embedding* can be constructed, in which each document is represented as a vector obtained by combining the vectors of the words that appear in the document. The goal is to be a vectorization such that documents on similar subjects are proximal. The words used in proximal documents will tend to have meanings that are related. Document embedding provides a framework for comparing documents and investigating the structure of a corpus.

Multidomain proteins poses challenges for bioinformatic techniques that use sequence analysis to extract functional, structural, and evolutionary information. In families with variable domain architectures, such as the MAGuKs, some sequence segments are homologous and have discernible sequence similarity; other regions are not alignable. As a result, bioinformatic tools based on sequence comparison cannot be directly applied to multidomain protein families with mosaic sequences. At the same time, the variations in domain composition carry information that current tools are not designed to exploit. Examining word context using vector semantics provides information about the relationships between words, and hence documents, that are independent of whether the words contain similar letters or are derived from the same root in an ancient language, suggesting the promise of this approach for multidomain protein architectures

Domain embeddings (Buchan and Jones 2020, Melidis and Nejdl 2021) have been used to investigate the potential of vector semantics for transferring functional information between neighboring domains. Moreover, domain embeddings have been shown to improve functional prediction of proteins (Ibtehaz *et al.* 2023, Wang *et al.* 2025). For instance, Ibtehaz et al. (2023) learned domain embeddings from domain-GO co-occurrence data, achieving state-of-the-art results on GO annotation tasks. Further integrating complementary features such as sequence similarity and protein interaction information enhanced prediction accuracy. These results support the view that domain composition carries meaningful functional signals.

### Our contributions

Restricting the study to a single genome results in a relatively small corpus. However, data sets that span multiple genomes have biases that are hard to characterize and correct. It is difficult to determine whether the same domain architecture, found in multiple related genomes, arose through convergent formation of that architecture in independent lineages or via vertical descent from the same domain architecture in a common ancestor. In the latter case, the multiple instances of the domain architecture represent observations of the same event and should be discarded. Uneven taxon sampling in the underlying database further exacerbates this problem. In addition, if the functional roles of the domains change over the course of evolution, combining information from distant genomes would further confound the signal.

A related problem arises with paralogs. If a series of domain gain and loss events gives rise to a gene encoding a multidomain architecture and that gene is subsequently duplicated, the resulting paralogs are not independent observations of the processes of multidomain evolution. To address this, we restrict our analysis to the set of unique domain architectures in the human proteome, which results in a single representative of each paralogous family with identical architectures. Any remaining paralogs pairs will have different domain architectures, e.g. through additional, post-duplication domain gains and/or losses, and hence do not reflect identical histories.

While the analogy between documents as strings of words and domain architectures as strings of domains is compelling, the characteristic scales of domain architecture data and natural language corpora are very different. In natural language corpora, sentences are typically 15 to 20 words in length, taken from a vocabulary of hundreds of thousands of words. Depending on the application, the document data available is almost unlimited. In contrast, the average length of a human domain architecture is less than 5 (median = 3), drawn from ∼1100 domains. There are approximately 5000 unique domain architectures in the human genome. With this in mind, we experimented with a number of domain embedding strategies, with modifications tuned to the scale of our data. We used two sparse embeddings, TF-IDF and Pointwise Mutual Information (PMI). For natural language applications, pointwise mutual information is typically restricted to positive values, focusing on words that co-occur more often than expected. However, with domain architectures, under-representation of domain pairs can be informative. Further, problems with underflow that occur with low frequency word pairs do not arise because of the small scale of DA data. We also experimented with Word2Vec using a skip-gram model, which accounts for local context; i.e. the domain order and content in a window of width 2w+1 centered on the current domain. In addition to the default window size of w=5, we experimented with a smaller window (w=1) to allow for the large number of domain architectures of length 5 or shorter. To account for the reduced scale of domain architecture data, we considered smaller dimensionalities (5 and 10), as well as the default value (100). We did not consider contextual embeddings (e.g. BERT representations, Devlin et al. (2018)), which account for contextual differences in meaning (e.g. homonyms) because most domain architectures are too short to benefit from that level of representational learning.

We apply this vector semantic framework to ask three questions about the relationship between domain content and protein function. First, do proteins with similar domain content also perform similar functions? To assess the association between domain architecture and protein function, for each embedding, we asked how accurately the function of a multidomain protein is predicted by the GO annotations of its neighbors in the embedding. Next, we asked whether this association is stronger when domain content similarity is assessed using vector embeddings, which carry implicit contextual information, compared with a direct comparison of protein domain content using Jaccard similarity, which provides a measure of the domains shared by two architectures, but does not use any of the additional information captured by a vector semantic embedding.

Third, we considered the case where neighboring domain architectures have no domains in common. In the natural language analogy, embeddings that capture words with similar or related meanings can address the “vocabulary problem”, where an information retrieval request fails because the word usage in the query does not match the word usage in the desired document (Furnas *et al.* 1987). We ask whether an analogous “domain vocabulary problem” exists for multidomain protein function and, if so, whether domain architecture embeddings can help to solve it.

Finally, we compared the domain architecture vector semantics with vector semantics based on domains only and observed a much weaker association between functional similarity and proximity in domain embeddings, compared with domain architecture embeddings. This may indicate that domain architecture embeddings encode more information, reflecting local context provided by flanking domains, to aid in accurate inference of functional contributions. Alternatively, the problem may be a lack of an appropriate ontology for describing domain function.

## Methods

### Dataset and pre-processing

Domain annotations (start and end positions) for 58 023 ENSEMBL protein sequences in the human genome (assembly GRCh38.p13) were downloaded from the SUPERFAMILY database, version 1.75 (Pandurangan *et al.* 2019). SUPERFAMILY uses a hierarchical classification based on SCOP, in which domains are grouped into families and superfamilies. Domains within the same superfamily share a structural core, although they may have low sequence similarity. In this work, we used the superfamily-level assignments. The pre-processing module of DomArchov (Cui *et al.* 2022) was used to extract the domain architectures of these sequences, resulting in a set of *N* = 5031 unique DAs, with an average length of 4.2 domains per DA. The median length is 3 and the max is 249. Overall, the length distribution is skewed toward shorter architectures: 4988 (99%) DAs have 30 or fewer domains. The distribution of DA length is shown in Supplementary Figure S1. These architectures comprise a set of 1109 distinct domain superfamilies, denoted D.

GO terms associated with the ENSEMBL protein sequences were obtained from ENSEMBL (Martin *et al.* 2023) and assigned to the corresponding domain architectures. Most DAs correspond to multiple ENSEMBL sequences. In these cases, the DA is annotated with those GO terms that are associated with at least 50% of all corresponding protein sequences. The list of GO associations is expanded to include the ancestors in the GO hierarchy connected by “is a” and “part of” relations. This resulted in 1157 distinct Molecular Function (MF), 1726 Biological Process (BP), and 378 Cellular Component (CC) GO terms assigned to human DAs. Of 5031 DAs, 4244 have at least one GO term. On average, each DA is mapped to 21 GO terms. Within the individual ontologies, 3910 DAs have at least one MF term; 2631 DAs have at least one BP term; 1295 DAs have at least one CC term. Any DA with only a protein-binding (GO: 0005515) annotation is removed from analyses for the MF ontology following the practice discussed in Zhou et al. (2019). The distribution of the number of GO terms per DA is shown in Supplementary Fig. S2.

### Domain embedding

Domain embeddings were constructed from the 5031 unique DAs using eight variants of three different vectorization strategies, where each domain $D_{i}\in D$ is represented as a vector, $e(D_{i})=[e_{1}(D_{i}),\ldots ,e_{m}(D_{i})]$. The dimensionality, *m*, depends on how the embedding is constructed.

#### Pointwise mutual information (PMI)

The pointwise mutual information (Church and Hanks 1990) of domain $D_{i}$ followed by $D_{j}$ is defined to be


(1) $$\text{PMI}(D_{i},D_{j})={\;\text{log}\;}_{2}\frac{P(D_{i},D_{j})}{P(D_{i})P(D_{j})},$$

where $P(D_{i})$ is the empirical frequency of domain superfamily $D_{i}$, and $P(D_{i},D_{j})$ is the empirical frequency of the ordered domain pair $D_{i}D_{j}$. A pseudocount ψ is added to the count of all ordered domain pairs. Following Cui et al. (2022), we use ψ=0.0009.

In the PMI embedding, the $j^{th}$ element of $e(D_{i})$ is $e_{j}(D_{i})=\text{PMI}(D_{i},D_{j})$. The dimensionality of this embedding is the number of unique domain superfamilies in the training data, m=|D|.

#### Term frequency-inverse document frequency (TF-IDF)

The inverse document frequency (Sparck Jones 1972) of $D_{i}$ is defined as $\text{idf}(D_{i})=N/\text{df}(D_{i})$, where $\text{df}(D_{i})$ is the number of DAs in which $D_{i}$ occurs and *N* is the total number of domain architectures in the dataset. For any domain $D_{i}$ and domain architecture $A_{j}$, the term frequency $\text{tf}(D_{i},A_{j})$ is the number of copies of $D_{i}$ in architecture $A_{j}$ (Luhn 1957). Here the $j^{th}$ element of $e(D_{i})$ is $e_{j}(D_{i})=\text{tf}(D_{i},A_{j})\cdot \text{idf}(D_{i})$ and m=N.

#### Word2vec

Word2Vec domain embeddings were constructed using the Python library gensim (Řehůřek and Sojka 2010) with the skip-gram model (Mikolov *et al.* 2013). Embeddings were calculated for vector sizes of m∈{5,10,100} and window sizes of w∈{1,5}, resulting in six different Word2Vec embeddings. Each model is later referred to by w2v(m,w). The models were each trained for 15 epochs.

### Domain architecture embedding

A domain architecture embedding was constructed for each of the eight domain embeddings (i.e TF-IDF, PMI, and 6 Word2Vec models) described above. Given domain architecture $A=D_{1}\ldots D_{n}$, the embedding vector of *A* is obtained by averaging the embeddings of its constituent domains


(2) $$v(A)=\frac{1}{n}\sum_{i}^{n}e(D_{i}).$$

Given a pair of domain architectures, $A_{i}$ and $A_{j}$, proximity in the embedding space is quantified using pairwise cosine similarity, denoted by $S_{C}(v(A_{i}),v(A_{j}))$.

The similarity of two architectures can also be quantified based on shared domain content, independent of an embedding, using the Jaccard similarity:


(3) $$S_{J}(A_{i},A_{j})=\frac{n_{ij}}{n_{i}+n_{j}-n_{ij}},$$

where $n_{ij}$ is the number of domain copies shared by $A_{i}$ and $A_{j}$. If $A_{i}$ and $A_{j}$ share no domains, then $S_{J}(A_{i},A_{j})=0$.

### Assessment

For assessments of functional similarity, only annotated multidomain architectures (i.e. DAs with 2 or more domains and at least one GO term) were considered. The numbers of annotated multidomain architectures in each of the three sub-ontologies are 3193 (MF), 2066 (BP), and 1001 (CC).

Let $A_{O}$ denote the set of annotated multidomain architectures in ontology O∈{MF,BP,CC}. Assessment centers around the functional attributes of the *k* nearest neighbors of a target domain architecture $A\in A_{O}$.

#### Annotated domain architecture neighborhoods

**Figure 2:**
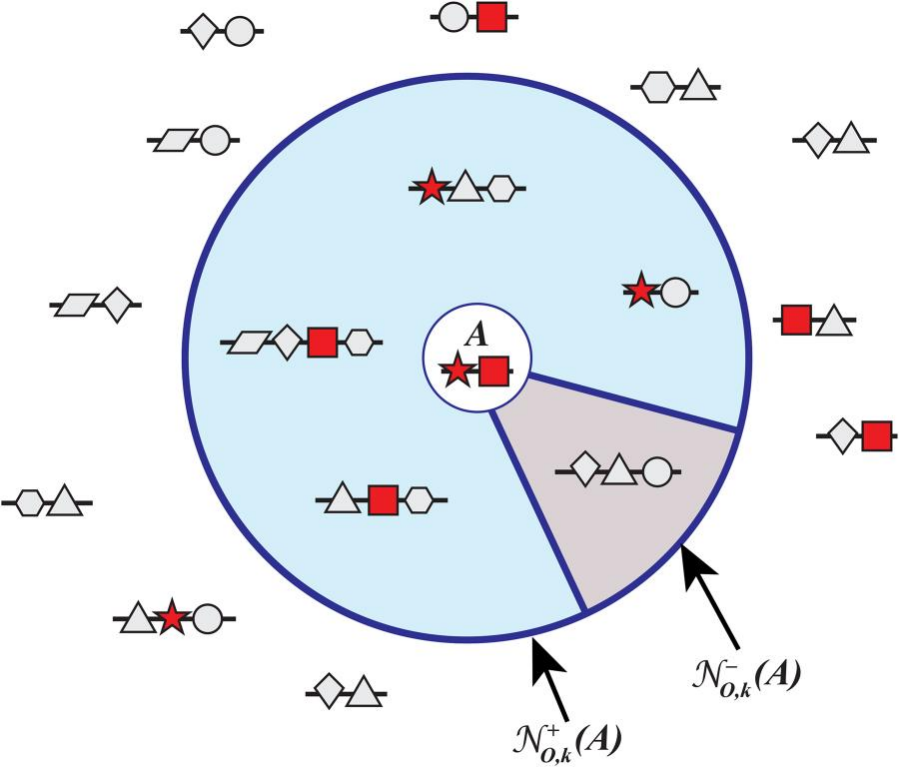
Example of a *k*-neighborhood with sharing and non-sharing architectures: The large circle outlined in a bold line shows the k=5 neighborhood of a target domain architecture, *A*. Domains in architecture *A* are shown as a star and rectangle. The sharing neighborhood of *A* (shaded, large wedge in circle) contains $k^{+}=4$ domain architectures that share at least one domain with *A*. The non-sharing neighborhood of *A* (smaller wedge) contains $k^{-}=1$ domain architecture. Note how $N_{O,k}(A)=N_{O,k}^{+}(A)\cup N_{O,k}^{-}(A)$.

Given an embedding *E*, let $N_{O,k}(A)$ be the annotated nearest neighborhood of size *k* for architecture *A*, separately for each of the three sub-ontologies *O*. We define this *k -neighborhood* to be the *k* DAs in $A_{O}$ that are closest to *A* in embedding *E* (Fig. 2).

Formally, $N_{O,k}(A)=\{A_{i_{1}}A_{i_{2}}\ldots A_{i_{k}}\},A_{i_{j}}\neq A$, is a *k*-neighborhood of *A* if for every $A_{i_{j}}$ in $N_{O,k}(A)$


$$S_{C}(v_{E}(A),v_{E}(A_{i_{j}}))>S_{C}(v_{E}(A),v_{E}(A_{b})),$$

for every $A_{b}$ that is not in $N_{O,k}(A)\cup \{A\}$. If there are two or more DAs that are equidistant from *A*, the k-neighborhood may not be unique. In this case, we assign one of the sets of *k* nearest neighbors to $N_{O,k}(A)$ arbitrarily. Additional analyses suggest that arbitrary tie breaking has negligible impact on the expected functional similarity of nearest neighbors (see assessment of alternative tie-breaking strategies in the supplementary text).

We further consider the subsets of $N_{O,k}(A)$: the *sharing k-neighborhood*, consisting of domain architectures that share at least one domain with *A*,


$$N_{O,k}^{+}(A)=\{A_{i}\in N_{O,k}(A)|S_{J}(A_{i},A)>0\}$$

and the *non-sharing k-neighborhood*, consisting of domain architectures that share no domains with *A*,


$$N_{O,k}^{-}(A)=\{A_{i}\in N_{O,k}(A)|S_{J}(A_{i},A)=0\}.$$

The respective sizes of these neighborhoods are $k^{+}$ and $k^{-}$, where $k^{+}+k^{-}=k$.

When comparing domain content in neighborhoods defined by Jaccard similarity, without reference to an embedding, the *k*-neighborhood of *A* is defined analogously. In this case, all DAs in the *k*-neighborhood of *A* have at least one domain in common with *A*, and thus $N_{O,k}^{+}(A)=N_{O,k}(A)$ and $N_{O,k}^{-}(A)=Ø.$

#### Functional similarity

##### Comparison of two domain architectures

For each ontology, let $F_{O}(A)$ denote the set of GO terms annotated to domain architecture *A*. The functional similarity between domain architectures $A_{i}$ and $A_{j}$, is defined to be the Jaccard similarity between $F_{O}(A_{i})$ and $F_{O}(A_{j})$:


(4) $$S_{F}^{O}(A_{i},A_{j})=\frac{\left|F_{O}(A_{i})\cap F_{O}(A_{j})\right|}{\left|F_{O}(A_{i})\cup F_{O}(A_{j})\right|}.$$

To avoid confusion due to use of the Jaccard similarity in two contexts, we use *functional similarity* to denote the similarity between two sets of GO terms and *Jaccard similarity* to refer to shared domain content (Eqn. 3).

##### Comparison of an architecture with its *k*-neighborhood

To assess whether domain architectures in the k-neighborhood of *A* have similar functions to *A*, we calculated $S_{F}^{O}(A,N_{O,k}(A))$, the functional similarity between *A* and its neighbors.

The GO term annotation of neighborhood $N_{O,k}(A)$ is defined to be the union of the GO terms associated with each architecture in the neighborhood set, $F_{O}(N_{O,k}(A))=\cup_{i_{j}}F_{O}(A_{i_{j}})$. The GO annotations of the sharing and non-sharing neighborhoods, $F_{O}(N_{O,k}^{+}(A))$ and $F_{O}(N_{O,k}^{-}(A))$, are defined analogously.

The functional similarity between architecture *A* and its neighborhood $N_{O,k}(A)$ is then defined as


(5) $$S_{F}^{O}(A,N_{O,k}(A))=\frac{\left|F_{O}(A)\cap F_{O}(N_{O,k}(A))\right|}{\left|F_{O}(A)\cup F_{O}(N_{O,k}(A))\right|}.$$

The functional similarity of architecture *A* to its sharing and non-sharing neighborhoods are denoted $S_{F}^{O}(A,N_{O,k}^{+}(A))$ and $S_{F}^{O}(A,N_{O,k}^{-}(A)),$ respectively.

For a given embedding and a given ontology, O∈{MF,BP,CC}, the mean functional similarity over all architectures in $A_{O}$,


$$\frac{1}{\left|A_{O}\right|}\sum_{A\in A_{O}}S_{F}^{O}(A,N_{O,k}(A))$$

provides a measure of how well that embedding places domain architectures with similar functions in close proximity. We quantify performance using precision, recall, and Matthews Correlation Coefficients (MCC).

##### Consistency within sharing *k*-neighborhoods

To assess how tightly focused sets of nearest neighbors are, we computed two measures for each sharing *k*-neighborhood. First, we considered the mean number of distinct GO terms in sharing *k*-neighborhoods, averaged across all target domain architectures,


(6) $$\sum_{A\in A_{O}}\frac{\left|F_{O}(N_{O,k}^{+}(A))\right|}{\left|A_{O}\right|}.$$

We also calculated the mean functional similarity within the sharing *k*-neighborhood obtained from all $\left(\begin{array}{c} k^{+}\\ 2 \end{array}\right)$ pairs of DAs in $N_{O,k}^{+}(A)$, averaged across all target domain architectures, that is,


(7) $$\sum_{A\in A_{O}}\frac{1}{\left|A_{O}\right|}\sum_{A_{1}\neq A_{2}\in N_{O,k}^{+}(A)}\frac{S_{F}^{O}(A_{1},A_{2})}{k^{+}(A)(k^{+}(A)-1)/2},$$

where $k^{+}(A)$ is the number of DAs in the sharing *k*-neighborhood of *A*.

#### Statistical tests for non-sharing nearest neighborhoods

We assessed the significance of observing neighboring domain architectures that are functionally similar, but have no common domains, using a randomization strategy with two test statistics.

Given an embedding and an ontology, let $A_{O}^{-}=\{A|N_{O,1}^{-}(A)\neq Ø\}$ be the set of architectures in $A_{O}$ that have at least one neighbor with no common domain. The first test statistic is the number of such pairs with functional similarity greater than 0.8:


(8) $$\sum_{A\in A_{O}^{-}}\frac{1}{\left|A_{O}^{-}\right|}\;\sum_{A^{\prime }\in N_{O,1}^{-}(A)}I(S_{F}^{O}(A,A^{\prime })>0.8),$$

where *I* is the indicator function.

The second is the mean functional similarity, averaged over all target architectures in $A_{O}^{-}$,


(9) $$\sum_{A\in A_{O}^{-}}\frac{1}{\left|A_{O}^{-}\right|}\;\sum_{A^{\prime }\in N_{O,1}^{-}(A)}S_{F}^{O}(A,A^{\prime }).$$

For each test statistic, the expected value was estimated from 100 000 null replicates generated as follows: for each target architecture in $A\in A_{O}^{-}$, a non-sharing partner DA was selected at random, with replacement, from the set of all DAs that share no domain with *A*. The value of the test statistic was calculated for set of null pairs generated in each replicate and averaged over all replicates to obtain the expected value. The empirical *p*-value is the proportion of replicates in which the null pairs achieve a greater value of the test statistic than the genuine pairs.

## Results

Here, we use vector semantics to investigate the relationship between protein domain architecture and protein function. We construct domain architecture embeddings using two sparse, high-dimensional encodings (TF-IDF, PMI) and six learned low-dimensional embeddings trained with Word2Vec. To better understand the underlying functional signals captured by embeddings, we next consider two scenarios, when proximal domain architectures share at least one domain, and when they do not have any domain in common. These cases allow us to decompose the contribution of domain content and learned contextual patterns in domain organization.

### Nearest neighbors that share at least one domain

First, we examine the scenario where nearest neighbors share at least one domain with the target. For each embedding, we ask: given a target domain architecture, how well do the combined GO terms of its *k* nearest annotated neighbors retrieve the GO annotations of the target? As a control, we calculate the same statistics using the *k* DAs with the highest Jaccard similarity to the target, that is, the *k* neighbors with the most similar domain content. This allows us to assess whether domain architecture embeddings provide more information than a simple domain content comparison.

We compared how well GO terms in sharing neighborhoods recapitulate the functional annotation of the target domain architecture in Table 1. For values of *k* greater than 1, the Word2Vec (m=5,w=5) embedding consistently obtained the best precision. The best recall was obtained when neighboring DAs were identified based on shared domain content using Jaccard similarity or TF-IDF, which also reflects shared domain content. This is also true when k=1 for the MF and BP ontologies (Table 1). In addition to GO term inheritance metrics, we examined the mean of the functional similarity between the target and its neighbors (Equation 5). For multidomain architectures that share at least one domain, neighboring domain architectures have more similar GO annotations when the neighborhood is determined using a DA embedding than when domain content alone is considered (Fig. S3, Table S3), except in the MF ontology when *k* is equal to one.

**Table 1:** Accuracy of GO annotation transfer in the sharing *k*-neighborhood for multidomain architectures as the mean $S_{F}^{O}(A,N_{O,k}^{+}(A))$ over all $A\in A_{O}$.

Molecular function	k=1			k=3			k=5			k=10		
	Precision	Recall	MCC	Precision	Recall	MCC	Precision	Recall	MCC	Precision	Recall	MCC
TF-IDF	**0.829**	0.816	0.806	0.671	*0.884*	0.745	0.580	0.907	0.695	0.455	0.932	0.615
PMI	0.789	0.796	0.778	0.651	0.874	0.732	0.573	0.898	0.69	0.471	0.921	0.625
w2v(100,5)	0.812	0.814	0.799	0.666	0.885	*0.746*	0.588	*0.905*	0.702	0.474	0.927	0.629
w2v(100,1)	0.792	0.793	0.778	0.649	0.869	0.728	0.570	0.894	0.686	0.475	0.917	0.626
w2v(10,5)	0.822	*0.818*	0.806	*0.677*	0.883	0.751	*0.596*	0.904	0.707	*0.490*	*0.924*	*0.640*
w2v(10,1)	0.809	0.804	0.792	0.669	0.877	0.743	0.588	0.896	0.699	0.488	0.918	0.636
w2v(5,5)	0.813	0.819	0.802	**0.702**	0.872	**0.762**	**0.636**	0.890	**0.728**	**0.542**	0.912	**0.673**
w2v(5,1)	0.792	0.793	0.777	0.679	0.853	0.739	0.618	0.872	0.707	0.540	0.898	0.665
Jaccard	*0.819*	**0.823**	**0.808**	0.643	**0.902**	0.736	0.530	**0.926**	0.668	0.382	**0.944**	0.559

Biological process	k=1			k=3			k=5			k=10		
	Precision	Recall	MCC	Precision	Recall	MCC	Precision	Recall	MCC	Precision	Recall	MCC
TF-IDF	**0.799**	**0.794**	**0.780**	0.644	0.859	**0.719**	0.546	0.883	0.662	0.415	0.910	0.576
PMI	0.741	0.747	0.727	0.591	0.832	0.673	0.513	0.863	0.631	0.413	0.891	0.565
w2v(100,5)	0.762	0.761	0.743	0.617	0.846	0.694	0.534	*0.873*	0.648	0.413	*0.899*	0.568
w2v(100,1)	0.741	0.740	0.723	0.605	0.837	0.683	0.526	0.861	0.638	0.422	0.889	0.572
w2v(10,5)	*0.773*	*0.776*	*0.758*	0.630	*0.847*	*0.703*	*0.547*	*0.873*	*0.658*	0.426	0.898	0.578
w2v(10,1)	0.757	0.759	0.741	0.619	0.842	0.694	0.545	0.870	0.655	*0.430*	0.895	*0.579*
w2v(5,5)	0.768	0.765	0.750	**0.653**	0.825	0.709	**0.589**	0.854	**0.679**	**0.486**	0.879	**0.616**
w2v(5,1)	0.755	0.759	0.738	*0.634*	0.817	0.691	0.570	0.833	0.656	0.481	0.869	0.607
Jaccard	0.792	0.787	0.772	0.604	**0.871**	0.698	0.487	**0.896**	0.626	0.310	**0.922**	0.494

Cellular component	k=1			k=3			k=5			k=10		
	Precision	Recall	MCC	Precision	Recall	MCC	Precision	Recall	MCC	Precision	Recall	MCC
TF-IDF	*0.891*	0.886	0.874	0.782	**0.926**	0.829	*0.710*	**0.939**	*0.790*	*0.611*	0.953	*0.727*
PMI	0.871	0.856	0.848	0.756	0.911	0.805	0.687	0.925	0.766	0.598	0.941	0.712
w2v(100,5)	0.886	0.879	0.868	0.756	0.922	0.808	0.695	*0.937*	0.776	0.593	0.948	0.710
w2v(100,1)	0.877	0.868	0.858	0.761	0.921	0.813	0.698	0.934	0.776	*0.611*	0.946	0.722
w2v(10,5)	0.888	0.886	0.874	*0.785*	0.924	0.829	0.703	*0.937*	0.782	0.603	*0.952*	0.720
w2v(10,1)	0.874	0.885	0.865	0.760	0.920	0.811	0.701	0.934	0.778	0.608	0.949	0.722
w2v(5,5)	**0.895**	**0.890**	**0.879**	**0.808**	0.921	**0.841**	**0.742**	0.932	**0.804**	**0.651**	0.941	**0.747**
w2v(5,1)	0.886	0.883	0.870	0.786	0.910	0.823	0.732	0.923	0.794	0.648	0.937	0.744
Jaccard	0.893	0.869	0.866	0.752	0.924	0.810	0.646	0.938	0.747	0.484	**0.955**	0.638

Best-performing method for each metric in bold, second-best is underlined, and third-best is italicized.

This led us to hypothesize that while shared domain content provides functional information, it is not sufficiently specific. To investigate this, we compared the number of distinct GO terms in the domain-sharing neighborhood (k=3 and k=5) obtained with Jaccard and with the various embeddings. The number of terms retrieved using Jaccard similarity exceeds the number of terms retrieved using all embeddings in all three ontologies, and is larger than the best embedding by as much as 50% (Table 2). Similarly, mean GO term similarity between domain architecture pairs within the domain-sharing neighborhood is consistently smaller with Jaccard, compared with all other methods (Table S4). These results suggest that shared domain content alone is not sufficiently precise. Embeddings capture more information than simply the presence or absence of shared domains.

**Table 2:** Functional consistency within sharing *k*-neighborhoods measured as the mean number of distinct GO terms in the sharing *k*-neighborhood (Eqn. 6).

Embedding	MF		BP		CC	
	k=3	k=5	k=3	k=5	k=3	k=5
TF-IDF	15.0	18.6	21.5	27.6	8.8	10.3
PMI	15.2	18.4	21.4	27.3	8.5	10.1
w2v(100,5)	15.1	18.4	21.9	28.0	9.3	10.9
w2v(100,1)	15.2	18.8	21.7	27.5	8.9	10.6
w2v(10,5)	14.6	18.0	21.2	27.2	8.7	10.5
w2v(10,1)	14.7	18.0	21.3	26.9	9.0	10.6
w2v(5,5)	12.5	15.3	16.7	21.0	7.4	8.9
w2v(5,1)	12.2	15.1	16.8	21.3	7.3	8.8
Jaccard	17.1	22.7	25.0	34.0	10.0	12.5

We wondered whether the number of domains in a domain architecture influences how well embeddings place the architecture into functionally similar neighborhoods. To assess the dependence of functional similarity on the lengths of the target and neighbor architectures, we applied ordinary least squares (OLS) linear regression to target-neighbor pairs from domain-sharing *k*-neighborhoods (k=1 and k=5). In all cases, $R^{2}$ values below 0.2 were obtained, suggesting little to no dependence on the number of domains in neighboring domain architectures (Figs. S4 and S5, Table S5). In addition, we applied similar analyses to the number of GO terms associated with neighboring domain architectures. Similarly, little to no dependence was observed, beyond the requirement of identical set sizes for maximal overlap (Figs. S6 and S7, Table S6).

### Nearest neighbors that share no domains

Embedding-based document similarity can identify texts on similar subjects even when they do not contain the same words. This is because they can contain words with similar or related meanings, captured by the embedding. By analogy, we ask whether embeddings are able to identify domain architectures with similar functions, even when they share no domains.

To explore this, we first asked how often multidomain architectures that share no domain are nearest neighbors in an embedding. Indeed, as many as 30% of proximal multidomain architectures have no domains in common (Table 3), depending on the embedding and the ontology. TF-IDF retrieves notably fewer such architectures than other embeddings, which is not surprising given that TF-IDF explicitly considers domains that are shared across architectures.

**Table 3:** Mean functional similarity ($S_{F}^{O}(A,N_{O,1}^{-}(A))$ averaged over all $A\in A_{O}$) in non-sharing neighborhoods (k=1).

	MF					BP					CC				
	# pairs^a^		Obs	Exp	*p*	# pairs^a^		Obs	Exp	*p*	# pairs^a^		Obs	Exp	*p*
TF-IDF	66	2%	0.117	0.219	0.834	68	3%	0.095	0.146	1.000	53	5%	0.406	0.311	**0.001**
PMI	210	7%	0.163	0.150	0.056	187	9%	0.162	0.143	0.024	114	11%	0.270	0.312	0.984
w2v(100,5)	149	4%	0.161	0.143	0.026	127	6%	0.187	0.146	**0.001**	89	9%	0.305	0.315	0.653
w2v(100,1)	187	5%	0.163	0.149	0.047	163	8%	0.177	0.148	**0.005**	106	10%	0.430	0.320	**0.000**
w2v(10,5)	200	6%	0.160	0.148	0.078	165	8%	0.174	0.146	**0.005**	100	10%	0.369	0.311	**0.005**
w2v(10,1)	198	6%	0.162	0.150	0.076	176	9%	0.182	0.148	**0.001**	99	10%	0.344	0.309	0.055
w2v(5,5)	637	20%	0.181	0.164	**0.001**	521	25%	0.186	0.153	**0.000**	223	22%	0.365	0.308	**0.000**
w2v(5,1)	752	23%	0.174	0.161	**0.002**	618	30%	0.190	0.154	**0.000**	268	27%	0.385	0.319	**0.000**

*P*-values below the 0.05 significance threshold after multiple hypothesis testing correction are shown in bold.

a The number and the percentage of DA-neighbor pairs that do not share domains.

**Figure 3:**
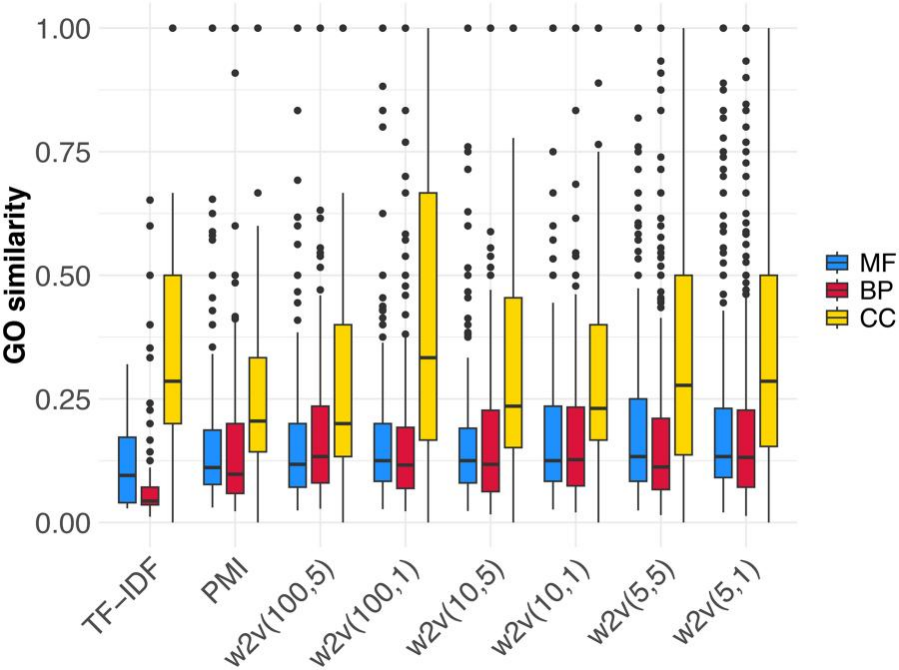
Functional similarity between target domain architectures and the k=1 nearest neighbor that lacks a common domain.

We next asked whether proximal domain architectures that share no domain with the target have similar GO terms (Fig. 3). For BP and CC, the majority of embeddings (shown in bold in Table 3) achieve significantly higher mean functional similarity than expected by chance. For MF, two embeddings identify non-sharing neighbors with higher than expected similarity. The observed mean similarity exceeds that of the null model for all embeddings except TF-IDF. Larger neighborhoods (k=3,5,10) tell a similar story: non-sharing neighbors remain highly functionally relevant (Fig. S8, Table S7).

To further assess whether embeddings identify non-sharing neighbors with meaningful similarities, we asked how often such pairs have high functional similarity. Indeed, embeddings consistently identify more pairs with functional similarity greater than 0.8 than expected by chance (Table 4). The results are statistically significant for w2v(5,1) in all three ontologies, for w2v(5,5) in BP and CC, and additionally w2v(100,1) and w2v(10,5) for CC.

**Table 4:** Number of neighboring multidomain pairs (k=1) that lack a common domain with functional similarity >0.8 (Eqn. 8).

	MF			BP		
	Obs	Exp	*p*	Obs	Exp	*p*
TF-IDF	0	0.1	0.134	0	0.3	0.298
PMI	2	0.9	0.062	6	1.2	**0.000**
w2v(100,5)	2	0.6	0.018	2	0.9	0.065
w2v(100,1)	3	0.8	0.007	4	1.5	0.018
w2v(10,5)	0	0.7	0.522	2	1.3	0.148
w2v(10,1)	0	0.7	0.519	4	1.4	0.012
w2v(5,5)	9	4.1	0.008	15	5.4	**0.000**
w2v(5,1)	11	4.4	**0.002**	15	6.7	**0.001**

	CC		
	Obs	Exp	*p*
TF-IDF	8	4.1	0.010
PMI	5	9.0	0.912
w2v(100,5)	8	7.7	0.356
w2v(100,1)	19	9.1	**0.000**
w2v(10,5)	15	7.8	**0.002**
w2v(10,1)	10	7.3	0.096
w2v(5,5)	26	16.4	**0.004**
w2v(5,1)	42	22.0	**0.000**

*P*-values below the 0.05 significance threshold after multiple hypothesis testing correction are shown in bold.

The possibility that domain architecture embeddings can identify multidomain pairs that share functional annotations, even when there is no overlapping domain content, is intriguing. To further explore this result, we examined the 187 unique pairs of nearest neighbors (k=1) that lack a shared domain, but have functional similarity above 0.8, identified by any embedding. Interestingly, only a small number of these 187 pairs are found by more than one embedding. This low overlap suggests that each embedding captures different contextual signals, highlighting complementary aspects of functional relationships between domain architectures. The complete list of such pairs is provided in Supplementary Information (S7). The threshold of 0.8 was selected to be consistent with the mean functional similarity observed in nearest neighbors (k=1) that share at least one domain (Table S3). The best threshold for identifying meaningful similarity is a question for future work.

It is possible that DA pairs that lack shared domains in our data set, but nevertheless have high functional similarity, do in fact share functional elements, but these elements are not in the Superfamily database. Functional elements that are not structural (e.g. transmembrane regions, localization signals) and as yet undiscovered domains are examples. To screen for such cases, we compared the amino acid sequences that correspond to the 187 pairs with functional similarity greater than 0.8, in search of conserved regions that might correspond to a shared functional element not represented in Superfamily. Out of 14 486 pairwise sequence comparisons (recall that most DAs correspond to more than one protein sequence) only 16 sequence pairs had local alignments with an *E* value less than 1. Only two domain architecture pairs corresponded to sequence comparisons with E values below 1 (*E* = 0.77 and *E* = 0.074, respectively). At time of writing, the default significance threshold in the blastp interface is *E* < 0.05.

**Figure 4:**
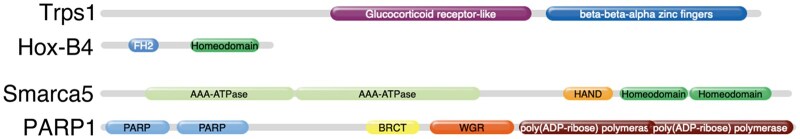
Schematic representation of domain architectures of two pairs of multidomain proteins that share no domains but have high functional term similarity (see text). The relative lengths of the proteins and domains are approximate.

We discuss two of these pairs in greater detail. The Zinc finger transcription factor Trps1 and Homeobox Hox-B4 proteins (Fig. 4, top panel) are both transcription factors involved in developmental regulation of the skeletal system (Kaiser *et al.* 2007, Dias *et al.* 2013). Trps1 is associated with regulation of chondrocyte differentiation (GO: 0032330), which is “part of” skeletal system development (GO: 0001501). Mutations in the gene are the basis of tricho-rhino-phalangeal syndrome type I (TRPS I) a genetic disorder characterized by skeletal abnormalities (Momeni *et al.* 2000). *HOXB4* is associated with embryonic skeletal system morphogenesis (GO: 0048704) and bone marrow development (GO: 0048539), also “part of” skeletal system development. *HOXB4* plays crucial roles in vertebrate skeletal system development (reviewed in Morgan et al. (2004)) and regulates the balance between differentiation into osteogenic (bone formation) or hematopoietic lineages of human embryonic stem cells (Kärner *et al.* 2009). As transcription factors, *TRPS1* and *HOXB4* also share GO terms such as regulation of RNA biosynthetic process (GO: 2001141). Both domain architectures contain DNA-binding domains (glucocorticoid receptor-like domains, zinc fingers, and homeodomains), but not the same DNA-binding domains. The blastp web interface identifies no significant similarity between these sequences at default settings.

SWI/SNF-related matrix-associated actin-dependent regulator of chromatin subfamily A member 5 (Smarca5) and Poly [ADP-ribose] polymerase (PARP) (Fig. 4, bottom panel) are DNA-binding proteins that participate in DNA repair (Toiber *et al.* 2013, Xie *et al.* 2015). They share CC GO terms (nucleolus (GO: 0005730) and site of double-strand break (GO: 0035861), and MF GO terms (DNA binding (GO: 0003677)). Both domain architectures encode DNA binding domains (HAND domains and homeodomains versus PARP zinc fingers and WGR domains), as well as domains with enzymatic activities (AAA-ATPases and poly(ADP-ribose)polymerases, respectively), but the specific domains in each of these categories differ in the two proteins. This pair has no significant sequence similarity at default parameter settings.

In this pilot study, vector embeddings identified neighboring domain architecture pairs that share functional properties but lack a common domain. There are more such pairs than expected by chance and the mean similarity of nearest neighbors that lack a shared domain is also greater than expected. However, many non-sharing nearest neighbors do not have strong functional similarity. Additional research is needed to determine how to identify the most promising pairs.

### Comparison with domain embeddings

We have seen that domain architectures that are proximal in DA embeddings also tend to be functionally similar. Since domain architecture embeddings are constructed from domain embeddings, we wondered whether there is a similar correlation between proximity and similarity in *domain* embeddings. We probed this question following the protocol of Buchan and Jones (2020), who constructed domain embeddings to investigate “the hypothesis that domains act as sub-functional units and when composed together, a protein’s given combination of domains is what gives rise to the protein’s overall specific function.” In the absence of a widely accepted domain-specific function ontology, Buchan and Jones (2020) annotated each domain with the set of all GO terms associated with proteins that harbor that domain. In order to compare domain architecture embedding results obtained in this paper with domain embedding results obtained by Buchan and Jones (2020), we assign to each domain the set of all GO terms associated with domain architectures that harbor that domain.

Buchan and Jones (2020) applied Word2Vec with default parameters (w=5,m=100) to PFAM domain architectures derived from ∼9 million eukaryotic proteins. We constructed a domain embedding with our human proteome data set and all eight embedding techniques used in the domain architecture embedding analysis. For each embedding, we calculated precision, recall, and MCC (Tables 5 and S8) to assess whether domains that are proximal in domain embeddings also have similar GO terms. For comparison, we calculated the same GO term inheritance metrics for full *k*-neighborhoods (including sharing and non-sharing neighbors) in domain architecture embeddings (Table S10).

**Table 5:** MCC values for GO annotation transfer in the *k*-neighborhood for domains, following the protocol of Buchan and Jones (2020).

	MF				BP				CC			
	k=1	k=3	k=5	k=10	k=1	k=3	k=5	k=10	k=1	k=3	k=5	k=10
TF-IDF	0.276	0.271	0.256	0.237	0.244	0.237	0.231	0.231	0.394	0.342	0.320	0.299
PMI	0.261	0.283	0.272	0.243	0.238	0.232	0.221	0.209	0.412	0.374	0.324	0.275
w2v(100,5)	0.259	0.270	0.264	0.234	0.225	0.218	0.208	0.187	0.412	0.378	0.350	0.289
w2v(100,1)	0.265	0.272	0.263	0.235	0.235	0.217	0.214	0.192	0.402	0.365	0.337	0.285
w2v(10,5)	0.266	0.278	0.267	0.235	0.230	0.226	0.210	0.193	0.416	0.373	0.343	0.281
w2v(10,1)	0.268	0.274	0.266	0.237	0.229	0.213	0.204	0.192	0.406	0.370	0.334	0.281
w2v(5,5)	0.267	0.272	0.265	0.241	0.239	0.224	0.216	0.193	0.405	0.363	0.336	0.283
w2v(5,1)	0.275	0.281	0.268	0.237	0.237	0.216	0.209	0.192	0.385	0.367	0.341	0.288
Buchan and Jones (2020)	0.300	0.230	0.220	0.190	0.270	0.200	0.190	0.170	0.330	0.220	0.220	0.200

The Matthews correlation coefficients we obtain (Table 5) are in close agreement with those obtained by Buchan and Jones (2020), despite the use of a different domain annotation database and substantial difference in data set sizes. These statistics do not support a strong relationship between functional similarity and proximity in domain embeddings. The MCC is less than 0.33 for all embeddings and all values of *k* tested. Similarly, meagre results are obtained when precision and recall are considered (Table S8). Moreover, we observe that GO term agreement within *k*-neighborhoods is much weaker in domain embeddings than in domain architecture embeddings. In all cases, the MCC obtained with domain embeddings is *less than half* that obtained with domain architecture embeddings.

These results suggest that proximity in a domain embedding provides relatively little information about the functional similarity of domains. However, functional similarity is assessed via GO term comparison and GO terms were designed to describe protein function, not domain function. It is difficult to determine whether domain embeddings do not encode domain function semantics, or whether GO terms are ill-suited to represent those semantics. As Buchan and Jones (2020) point out, a domain-centric functional ontology is needed in order to resolve this question.

## Discussion

Mounting evidence suggests that protein domains carry information about the functions of the proteins that encode them, but explicit models that relate domain content to protein function are lacking. In particular, we require a better understanding of how the functional relevance of a particular domain depends on its surrounding context. Here, we investigated the potential of vector semantic embeddings to capture contextual information. We observe that pairs of multidomain architectures identified using vector semantics share more functional attributes than pairs of multidomain architectures selected based on domain content similarity, alone. Our results suggest that these vector semantic models may capture combinatorial interactions of domains in the same protein. Surprisingly, in some cases, vector semantic embeddings identified multidomain architecture pairs that had high functional similarity, *despite having no domains in common at all*. Note that no information from sequence analysis or biological textual descriptions were used to construct the embeddings; these inferences are based on domain co-occurrence alone.

Taken together, these results underscore the potential of protein domain vector semantics to elucidate the “design rules” of multidomain architectures. One hypothesis for multidomain protein function is that domain functions are additive. Under this “functional decomposition” hypothesis, the function of a domain architecture is the sum of its parts. An alternate hypothesis is that the functional contribution of a domain depends on the broader context of other domains in the architecture. Our results suggest that the functional decomposition hypothesis may be too simple. If domains perform different functional roles in different proteins, then additional contextual information could be required to determine which functional role is most relevant. This explanation is consistent with our observation that domain architecture embeddings better predict functional similarity than shared domain content; domain architecture embeddings may be providing the required contextual information. Similarly, this may explain why domain embeddings are less effective than domain architecture embeddings in predicting shared function, since domain architecture embeddings incorporate additional contextual information not represented in domain embeddings. Finally, it is also consistent with the lack of correlation between the functional similarity of a pair of domain architectures and the number of domains that they contain. If domain functions were additive, we would expect to see a length effect: the longer the domain architecture, the smaller the contribution of any one domain. However, no such length effect is observed.

While intriguing, these results are preliminary. Further investigations that comprehend larger data sets and greater taxonomic breadth are required. In addition, the revolution in protein structure prediction driven by advances in deep learning has produced greatly expanded bioinformatic resources for protein domain analyses (Lau *et al.* 2024, Paysan-Lafosse *et al.* 2025). Future work that incorporates this new knowledge is also imperative.

The results presented here suggest that protein domain vector semantics may be able to discover the protein domain equivalent of words and phrases with similar or related meanings in natural languages. The science of lexical semantics deals with many types of related meanings (reviewed in Jurafsky and Martin 2008). Words may have similar meanings (e.g. *sarcastic*, *ironic*), be used in the same context (e.g. *coffee*, *cup*), be associated with the same activity (e.g. *suture*, *scalpel*), or describe objects that are made of the same materials or have similar constituent parts (e.g. *car*, *bicycle*). Characterizing different types of word associations and developing NLP models that are capable of distinguishing between them are active areas of NLP research (Hill *et al.* 2015). Our discovery of functionally similar multidomain architectures that harbor no common domains suggests that notions of semantic or topical relatedness may be relevant to protein domain function. What types of “topical relatedness” are meaningful in the protein domain context is an exciting open question. In one of the first works to tackle this question, Buchan and Jones (2020) experimented with vector arithmetic models of word analogies (e.g. *king* is to *man* as *queen* is to *woman*) to explore whether such relations also exist for domains.

Vector semantic models of multidomain proteins also hold promise for advancing bioinformatic applications. The problem of developing a *Domain Ontology* is closely related to questions in protein domain lexical semantics. The Gene Ontology (GO) is a powerful and general system for describing protein function. The hierarchy of well-defined functional terms using a controlled vocabulary supports a broad range of applications to query, manipulate, compare and reason with biological data. While several lines of research are advancing functional annotation for domains (Weiner *et al.* 2008, Burge *et al.* 2012, Fang and Gough 2013, López and Pazos 2013, Buchan and Jones 2020, Blum *et al.* 2021, Ibtehaz *et al.* 2023, Ulusoy and Doğan 2024), no equivalent domain function ontology currently exists. Nor is there agreement on what a domain-specific ontology should look like. Should domain ontology terms describe inherent functional properties of the domains themselves? Or is the goal to annotate domains with terms that predict the functions of any protein that is found to encode the domain? Several lines of research are advancing functional annotation for domains. It may be possible to partition the molecular functions of a protein by attributing specific molecular roles to domains, but it is less clear whether protein domains tend to be associated with specific biological processes or cellular compartments. This is an interesting research question that has practical consequences for the development of bioinformatic resources.

Vector semantics of domains and domain architectures also holds promise for *protein function prediction* (Radivojac *et al.* 2013, Jiang *et al.* 2016, Zhou *et al.* 2019). A better understanding of multidomain design rules will shed light on the predictive value of domain content and organization for multidomain protein functions. This is important: In the first Critical Assessment of protein Function Annotation (CAFA) challenge, all of the top predictors performed less well on multidomain proteins than on single domain proteins (Radivojac *et al.* 2013). Protein function prediction methods are increasingly combining many different types of data (Radivojac *et al.* 2013, Jiang *et al.* 2016, Zhou *et al.* 2019, Boadu *et al.* 2025), including domain family information, but few methods have attempted to incorporate an explicit model relating domain content to the functional attributes of multidomain proteins. The success of several notable exceptions (You *et al.* 2018, Ibtehaz *et al.* 2023, Talo and Bozdag 2025) suggests the value of vector semantics in this context.

## Supplementary Material

vbag037_Supplementary_Data

## Data Availability

The data and code used in this work are available at https://zenodo.org/records/15769961.
