# Supplementary material for: Vector semantics of multidomain protein architectures
Source: Bioinform Adv. 2026 Mar 2;6(1):vbag037. doi: 10.1093/bioadv/vbag037 (PMC13049198; doi:10.1093/bioadv/vbag037)
Supplement: vbag037_Supplementary_Data [file vbag037_supplementary_data.pdf]

Supplementary information for
Vector Semantics of Multidomain Protein Architectures

Statistics of dataset

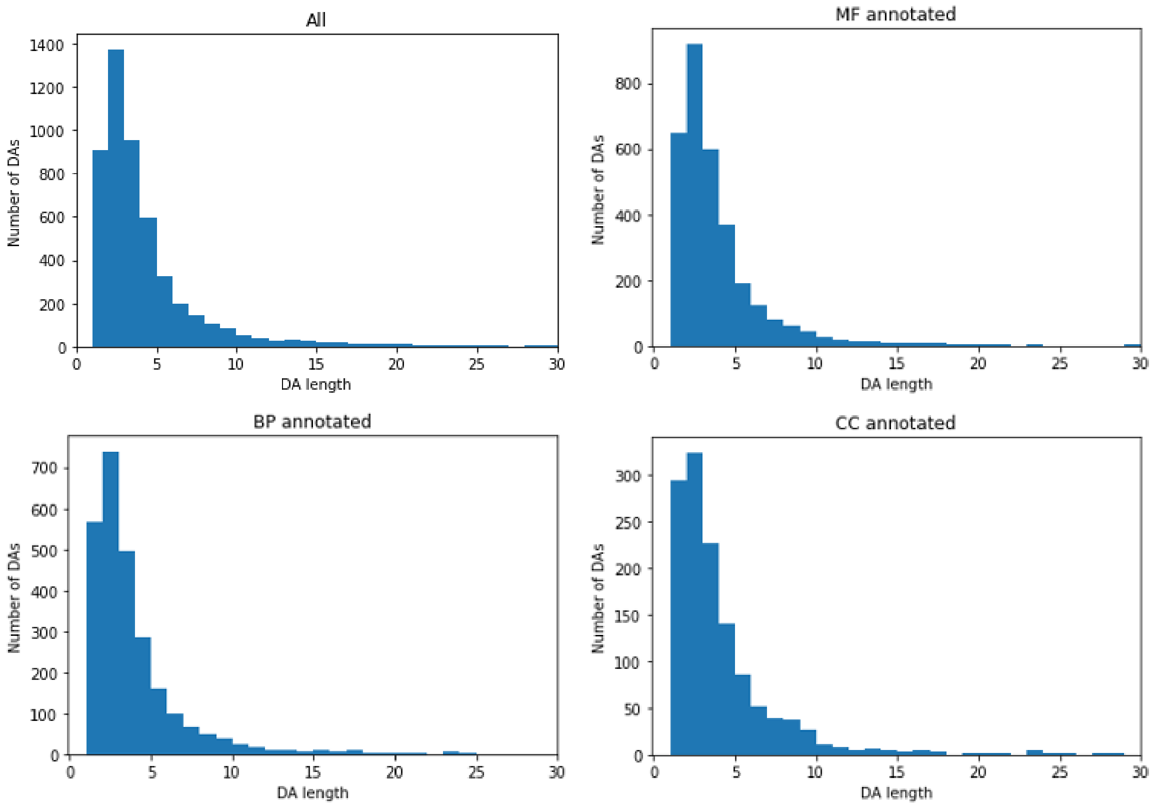

Fig. S1. Distribution of lengths of all domain architectures, and domain architectures with MF, BP, and CC annotations. For clarity, each histogram is shown only for lengths 1-30. Domain architectures longer than 30 domains account for only 1% of all cases.

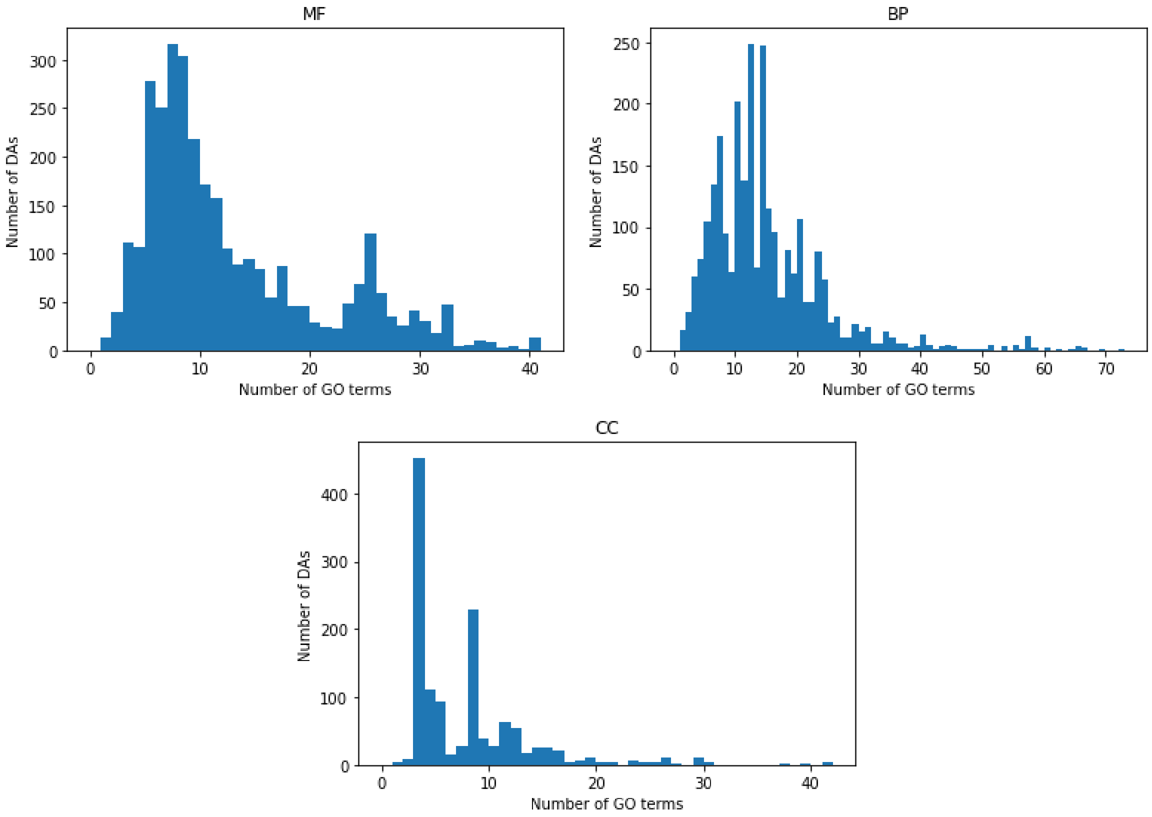

Fig. S2. Distribution of the number of GO terms associated with domain architectures in each sub-ontology.

Assessment of alternative tie-breaking strategies

In our datasets, distance ties are relatively uncommon (in any given experiment, $\approx 10\%$ of query architectures result in a tie). We assessed the impact of tie handling with two different strategies:

1. For every query with a tie, we replaced the arbitrarily chosen neighbor(s) with a different tied neighbor(s). Across all models, sub-ontologies, and values of k, the resulting changes in precision, recall, and MCC were negligible (absolute differences≤ 0.003, Table S1).
2. We expanded the neighborhood to include all items at the tied boundary (i.e., increased the effective k until the next strictly more distant neighbor). This resulted in a modest performance decrease for small neighborhoods (about 0.01 absolute for $k \in \{1, 3\}$), and a smaller change for larger neighborhoods ($k \in \{5, 10\}$), consistent with the intuition that bigger values of k are less sensitive to the choice (Table S2).

These results indicate that the random tie-breaking used in the main analyses does not substantially affect the conclusions.

Table S1. Accuracy of GO annotation transfer in the sharing k-neighborhood for multidomain architectures as the mean $S_F^O(A, \mathcal{N}_{O,k}^+(A))$ over all $A \in \mathcal{A}_O$, obtained with an alternative tie-breaking strategy where for every query with a tie, the arbitrarily chosen neighbor(s) is replaced with a different tied neighbor(s).

**Molecular Function**	$k=1$			$k=3$			$k=5$			$k=10$		
	precision	recall	MCC	precision	recall	MCC	precision	recall	MCC	precision	recall	MCC
TF-IDF	0.830	0.816	0.807	0.672	0.885	0.746	0.580	0.907	0.695	0.454	0.932	0.615
PMI	0.789	0.794	0.778	0.650	0.876	0.732	0.572	0.898	0.690	0.472	0.921	0.625
w2v(100,5)	0.812	0.814	0.799	0.667	0.886	0.746	0.587	0.906	0.702	0.474	0.927	0.629
w2v(100,1)	0.793	0.793	0.779	0.649	0.870	0.728	0.570	0.894	0.686	0.475	0.917	0.626
w2v(10,5)	0.821	0.817	0.805	0.679	0.884	0.753	0.596	0.904	0.708	0.490	0.924	0.640
w2v(10,1)	0.811	0.804	0.793	0.667	0.875	0.742	0.589	0.896	0.699	0.488	0.918	0.636
w2v(5,5)	0.812	0.817	0.801	0.703	0.872	0.762	0.636	0.890	0.728	0.542	0.912	0.673
w2v(5,1)	0.790	0.794	0.776	0.680	0.853	0.739	0.617	0.871	0.707	0.539	0.898	0.665

**Biological process**	$k=1$			$k=3$			$k=5$			$k=10$		
	precision	recall	MCC	precision	recall	MCC	precision	recall	MCC	precision	recall	MCC
TF-IDF	0.801	0.793	0.781	0.641	0.858	0.716	0.547	0.882	0.663	0.415	0.91	0.576
PMI	0.744	0.748	0.729	0.589	0.83	0.671	0.513	0.862	0.631	0.414	0.891	0.565
w2v(100,5)	0.761	0.761	0.744	0.619	0.847	0.696	0.533	0.872	0.647	0.413	0.899	0.568
w2v(100,1)	0.747	0.740	0.726	0.604	0.837	0.683	0.527	0.860	0.639	0.423	0.890	0.573
w2v(10,5)	0.775	0.774	0.758	0.632	0.846	0.703	0.545	0.874	0.657	0.426	0.898	0.578
w2v(10,1)	0.760	0.764	0.744	0.617	0.843	0.693	0.544	0.870	0.654	0.430	0.896	0.580
w2v(5,5)	0.764	0.765	0.747	0.655	0.828	0.711	0.590	0.853	0.679	0.486	0.880	0.616
w2v(5,1)	0.755	0.755	0.736	0.637	0.819	0.694	0.569	0.833	0.655	0.482	0.869	0.607

**Cellular component**	$k=1$			$k=3$			$k=5$			$k=10$		
	precision	recall	MCC	precision	recall	MCC	precision	recall	MCC	precision	recall	MCC
TF-IDF	0.893	0.886	0.876	0.780	0.925	0.828	0.709	0.939	0.789	0.612	0.953	0.728
PMI	0.871	0.866	0.853	0.757	0.911	0.805	0.688	0.925	0.767	0.599	0.941	0.713
w2v(100,5)	0.878	0.880	0.863	0.758	0.922	0.810	0.695	0.937	0.776	0.593	0.948	0.710
w2v(100,1)	0.878	0.872	0.861	0.760	0.921	0.812	0.699	0.934	0.777	0.612	0.945	0.723
w2v(10,5)	0.888	0.884	0.872	0.783	0.924	0.828	0.707	0.936	0.784	0.603	0.953	0.720
w2v(10,1)	0.876	0.883	0.865	0.761	0.921	0.812	0.698	0.934	0.777	0.606	0.949	0.721
w2v(5,5)	0.897	0.889	0.879	0.805	0.921	0.838	0.745	0.933	0.806	0.651	0.942	0.747
w2v(5,1)	0.892	0.877	0.870	0.785	0.910	0.822	0.733	0.922	0.794	0.649	0.937	0.745

Table S2. Accuracy of GO annotation transfer in the sharing k-neighborhood for multidomain architectures as the mean $S_F^O(A, \mathcal{N}_{O,k}^+(A))$ over all $A \in \mathcal{A}_O$, obtained with an alternative tie-breaking strategy where the neighborhood is expanded to include all items at the tied boundary (i.e., increasing the effective k until the next strictly more distant neighbor).

**Molecular Function**	$k = 1$			$k = 3$			$k = 5$			$k = 10$		
	precision	recall	MCC	precision	recall	MCC	precision	recall	MCC	precision	recall	MCC
TF-IDF	0.821	0.822	0.806	0.665	0.885	0.742	0.576	0.908	0.693	0.453	0.932	0.614
PMI	0.786	0.800	0.779	0.648	0.874	0.731	0.572	0.898	0.690	0.471	0.921	0.625
w2v(100,5)	0.806	0.821	0.799	0.664	0.887	0.745	0.587	0.906	0.702	0.474	0.927	0.629
w2v(100,1)	0.788	0.797	0.778	0.647	0.870	0.728	0.569	0.894	0.686	0.475	0.917	0.626
w2v(10,5)	0.816	0.824	0.806	0.675	0.884	0.750	0.594	0.904	0.706	0.490	0.924	0.640
w2v(10,1)	0.805	0.809	0.793	0.666	0.877	0.742	0.587	0.896	0.698	0.488	0.918	0.636
w2v(5,5)	0.809	0.825	0.803	0.701	0.873	0.761	0.635	0.890	0.727	0.541	0.912	0.673
w2v(5,1)	0.786	0.801	0.777	0.678	0.854	0.738	0.617	0.872	0.707	0.539	0.898	0.665

**Biological process**	$k = 1$			$k = 3$			$k = 5$			$k = 10$		
	precision	recall	MCC	precision	recall	MCC	precision	recall	MCC	precision	recall	MCC
TF-IDF	0.790	0.802	0.780	0.637	0.863	0.715	0.542	0.884	0.660	0.414	0.910	0.575
PMI	0.734	0.756	0.726	0.586	0.834	0.670	0.512	0.864	0.630	0.412	0.891	0.564
w2v(100,5)	0.749	0.772	0.741	0.610	0.851	0.692	0.529	0.874	0.646	0.413	0.899	0.568
w2v(100,1)	0.732	0.749	0.721	0.599	0.840	0.681	0.520	0.862	0.635	0.422	0.890	0.572
w2v(10,5)	0.765	0.783	0.756	0.624	0.850	0.701	0.542	0.874	0.655	0.424	0.898	0.577
w2v(10,1)	0.749	0.769	0.740	0.615	0.844	0.692	0.542	0.870	0.653	0.428	0.895	0.578
w2v(5,5)	0.753	0.777	0.746	0.649	0.831	0.708	0.585	0.856	0.677	0.484	0.879	0.615
w2v(5,1)	0.745	0.768	0.736	0.633	0.821	0.692	0.568	0.834	0.655	0.480	0.869	0.606

**Cellular component**	$k = 1$			$k = 3$			$k = 5$			$k = 10$		
	precision	recall	MCC	precision	recall	MCC	precision	recall	MCC	precision	recall	MCC
TF-IDF	0.888	0.887	0.872	0.778	0.927	0.827	0.705	0.940	0.787	0.610	0.953	0.726
PMI	0.860	0.867	0.847	0.753	0.912	0.803	0.686	0.926	0.766	0.598	0.941	0.712
w2v(100,5)	0.874	0.882	0.862	0.756	0.922	0.808	0.693	0.937	0.775	0.593	0.948	0.710
w2v(100,1)	0.864	0.877	0.855	0.750	0.924	0.807	0.686	0.936	0.771	0.610	0.947	0.722
w2v(10,5)	0.875	0.890	0.867	0.772	0.926	0.822	0.695	0.938	0.777	0.602	0.952	0.719
w2v(10,1)	0.867	0.887	0.862	0.759	0.921	0.811	0.696	0.934	0.775	0.605	0.949	0.720
w2v(5,5)	0.880	0.895	0.872	0.796	0.923	0.834	0.736	0.934	0.801	0.650	0.941	0.746
w2v(5,1)	0.880	0.885	0.867	0.782	0.911	0.821	0.731	0.923	0.793	0.647	0.937	0.743

Nearest neighbors that share at least one domain

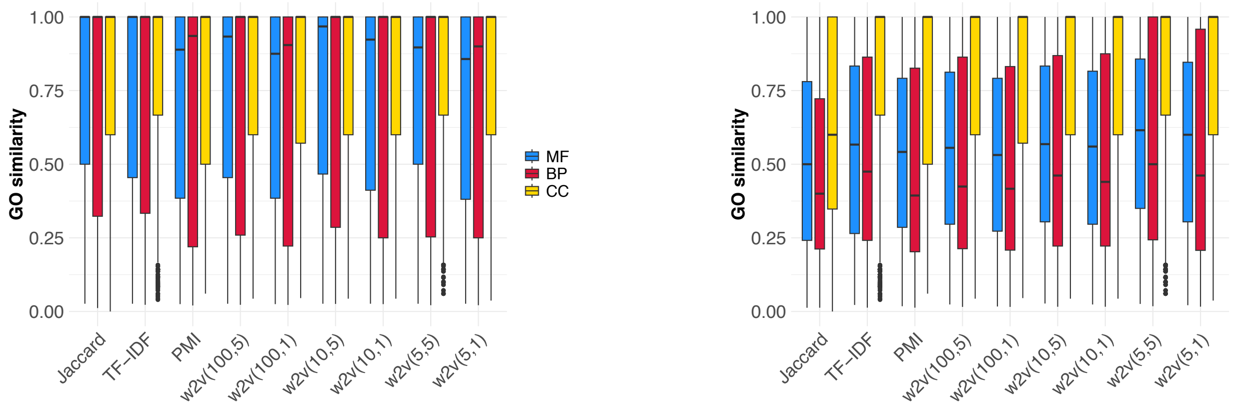

Fig. S3. Functional similarity between target domain architectures and the nearest neighbors when $k = 1$ (left) or $k = 5$ (right) that share at least one domain. GO terms associated with each target are compared to the union of GO terms from neighbors.

Table S3. Mean functional similarity within sharing k-neighborhoods measured as $S_F^O(A, \mathcal{N}^+{}_{O,k}(A))$ averaged over all $A \in \mathcal{A}_O$.

MF	$k = 1$	$k = 3$	$k = 5$	$k = 10$
TF-IDF	0.753	0.657	0.576	0.436
PMI	0.717	0.622	0.548	0.442
w2v(100,5)	0.746	0.649	0.560	0.447
w2v(100,1)	0.724	0.624	0.542	0.439
w2v(10,5)	0.752	0.661	0.574	0.466
w2v(10,1)	0.736	0.642	0.566	0.457
w2v(5,5)	0.740	0.659	0.601	0.507
w2v(5,1)	0.725	0.642	0.580	0.497
Jaccard	0.754	0.637	0.524	0.375

BP	$k = 1$	$k = 3$	$k = 5$	$k = 10$
TF-IDF	0.724	0.613	0.526	0.403
PMI	0.662	0.559	0.492	0.399
w2v(100,5)	0.680	0.583	0.511	0.399
w2v(100,1)	0.657	0.570	0.501	0.406
w2v(10,5)	0.699	0.595	0.524	0.411
w2v(10,1)	0.678	0.585	0.522	0.415
w2v(5,5)	0.689	0.608	0.557	0.464
w2v(5,1)	0.672	0.588	0.534	0.459
Jaccard	0.717	0.584	0.476	0.305

CC	$k = 1$	$k = 3$	$k = 5$	$k = 10$
TF-IDF	0.827	0.749	0.686	0.596
PMI	0.794	0.718	0.659	0.579
w2v(100,5)	0.820	0.719	0.668	0.573
w2v(100,1)	0.807	0.725	0.670	0.590
w2v(10,5)	0.826	0.750	0.677	0.586
w2v(10,1)	0.816	0.724	0.674	0.589
w2v(5,5)	0.833	0.767	0.710	0.623
w2v(5,1)	0.821	0.742	0.695	0.621
Jaccard	0.817	0.722	0.628	0.476

Functional consistency within sharing k-neighborhoods

Table S4. Average pairwise GO similarity between neighbors in neighborhood of size k, excluding the target domain architecture (Eqn. 7).

	MF		BP		CC	
Embedding	$k=3$	$k=5$	$k=3$	$k=5$	$k=3$	$k=5$
TF-IDF	0.68	0.64	0.61	0.56	0.76	0.73
PMI	0.65	0.61	0.57	0.54	0.75	0.72
w2v(100,5)	0.67	0.63	0.59	0.55	0.74	0.72
w2v(100,1)	0.64	0.61	0.57	0.54	0.73	0.72
w2v(10,5)	0.69	0.64	0.61	0.57	0.77	0.74
w2v(10,1)	0.67	0.64	0.59	0.56	0.74	0.72
w2v(5,5)	0.69	0.66	0.65	0.61	0.78	0.75
w2v(5,1)	0.67	0.64	0.61	0.59	0.74	0.73
Jaccard	0.61	0.57	0.54	0.49	0.70	0.67

Dependence of neighborhood inheritance on DA length and the number of GO terms

Table S5. Results of linear regression assessing whether DA length predicts functional similarity. Linear model: $y = \beta_0 + \beta_1 x_1 + \beta_2 x_2$, where y is functional similarity between the target DA and its domain-sharing k-nearest neighbor, x_1 is the length of the target domain architecture (the number of domains), and x_2 is the length of the neighbor DA.

	MF				BP				CC			
$k=1$	R^2	β_0	β_1	β_2	R^2	β_0	β_1	β_2	R^2	β_0	β_1	β_2
TF-IDF	0.010	0.721	0.003	0.001	0.009	0.710	0.004	-0.001	0.017	0.800	0.010	-0.004
PMI	0.013	0.688	0.005	0.000	0.010	0.640	0.003	0.001	0.029	0.741	0.006	0.004
w2v(100,5)	0.012	0.712	0.005	-0.001	0.008	0.661	0.003	0.001	0.022	0.775	0.006	0.003
w2v(100,1)	0.013	0.686	0.005	-0.001	0.009	0.637	0.003	0.001	0.021	0.763	0.007	0.002
w2v(10,5)	0.011	0.725	0.005	-0.002	0.006	0.683	0.003	0.001	0.019	0.786	0.005	0.003
w2v(10,1)	0.011	0.703	0.004	0.000	0.007	0.661	0.003	0.001	0.018	0.777	0.007	0.001
w2v(5,5)	0.010	0.717	0.004	-0.001	0.007	0.672	0.002	0.001	0.016	0.797	0.005	0.001
w2v(5,1)	0.016	0.685	0.006	-0.002	0.008	0.654	0.003	0.000	0.018	0.785	0.007	-0.001
	MF				BP				CC			
$k=5$	R^2	β_0	β_1	β_2	R^2	β_0	β_1	β_2	R^2	β_0	β_1	β_2
TF-IDF	0.010	0.584	0.004	0.001	0.008	0.557	0.003	0.001	0.012	0.706	0.007	-0.001
PMI	0.011	0.586	0.003	0.001	0.006	0.539	0.002	0.001	0.019	0.674	0.007	0.001
w2v(100,5)	0.009	0.603	0.003	0.002	0.007	0.552	0.002	0.002	0.015	0.691	0.005	0.002
w2v(100,1)	0.008	0.585	0.002	0.002	0.007	0.541	0.003	0.001	0.010	0.687	0.003	0.003
w2v(10,5)	0.007	0.612	0.003	0.001	0.004	0.570	0.002	0.001	0.013	0.700	0.004	0.003
w2v(10,1)	0.009	0.597	0.003	0.002	0.006	0.559	0.002	0.002	0.016	0.682	0.005	0.004
w2v(5,5)	0.004	0.635	0.001	0.001	0.003	0.599	0.001	0.001	0.011	0.723	0.003	0.004
w2v(5,1)	0.005	0.613	0.002	0.001	0.005	0.578	0.002	0.001	0.003	0.719	0.001	0.002

Table S6. Results of linear regression assessing whether the number of associated GO terms predicts functional similarity. Linear model: $y = \beta_0 + \beta_1 x_1 + \beta_2 x_2$, where y is functional similarity between the target DA and its domain-sharing k-nearest neighbor, x_1 is the number of GO terms the target domain architecture is associated with, and x_2 is the number of GO terms the neighbor is associated with.

	MF				BP				CC			
$k = 1$	R^2	β_0	β_1	β_2	R^2	β_0	β_1	β_2	R^2	β_0	β_1	β_2
TF-IDF	0.009	0.689	0.001	0.003	0.008	0.780	-0.003	-0.001	0.046	0.912	-0.010	-0.002
PMI	0.013	0.653	0.006	-0.002	0.021	0.767	-0.003	-0.004	0.087	0.922	-0.015	-0.004
w2v(100,5)	0.006	0.689	0.001	0.002	0.026	0.796	-0.005	-0.003	0.079	0.938	-0.010	-0.007
w2v(100,1)	0.012	0.643	0.002	0.003	0.020	0.754	-0.005	-0.002	0.092	0.939	-0.010	-0.009
w2v(10,5)	0.007	0.697	0.000	0.003	0.026	0.812	-0.003	-0.005	0.060	0.922	-0.007	-0.007
w2v(10,1)	0.008	0.674	0.001	0.003	0.025	0.793	-0.005	-0.003	0.078	0.934	-0.006	-0.011
w2v(5,5)	0.005	0.709	0.004	-0.002	0.020	0.783	-0.005	-0.002	0.072	0.943	-0.010	-0.006
w2v(5,1)	0.007	0.655	0.002	0.002	0.027	0.793	-0.004	-0.004	0.101	0.949	-0.012	-0.007

	MF				BP				CC			
$k = 5$	R^2	β_0	β_1	β_2	R^2	β_0	β_1	β_2	R^2	β_0	β_1	β_2
TF-IDF	0.005	0.564	0.002	0.001	0.020	0.671	-0.006	0.000	0.067	0.857	-0.011	-0.006
PMI	0.014	0.539	0.004	0.001	0.027	0.686	-0.004	-0.005	0.097	0.872	-0.012	-0.010
w2v(100,5)	0.013	0.558	0.004	0.001	0.028	0.700	-0.005	-0.004	0.091	0.874	-0.010	-0.010
w2v(100,1)	0.011	0.544	0.004	0.001	0.034	0.702	-0.005	-0.005	0.103	0.873	-0.009	-0.012
w2v(10,5)	0.011	0.567	0.003	0.002	0.026	0.708	-0.004	-0.004	0.082	0.871	-0.009	-0.009
w2v(10,1)	0.011	0.554	0.002	0.003	0.025	0.697	-0.005	-0.004	0.111	0.884	-0.011	-0.012
w2v(5,5)	0.006	0.603	0.002	0.001	0.024	0.722	-0.005	-0.003	0.075	0.883	-0.009	-0.009
w2v(5,1)	0.009	0.573	0.003	0.001	0.033	0.729	-0.004	-0.005	0.119	0.898	-0.014	-0.011

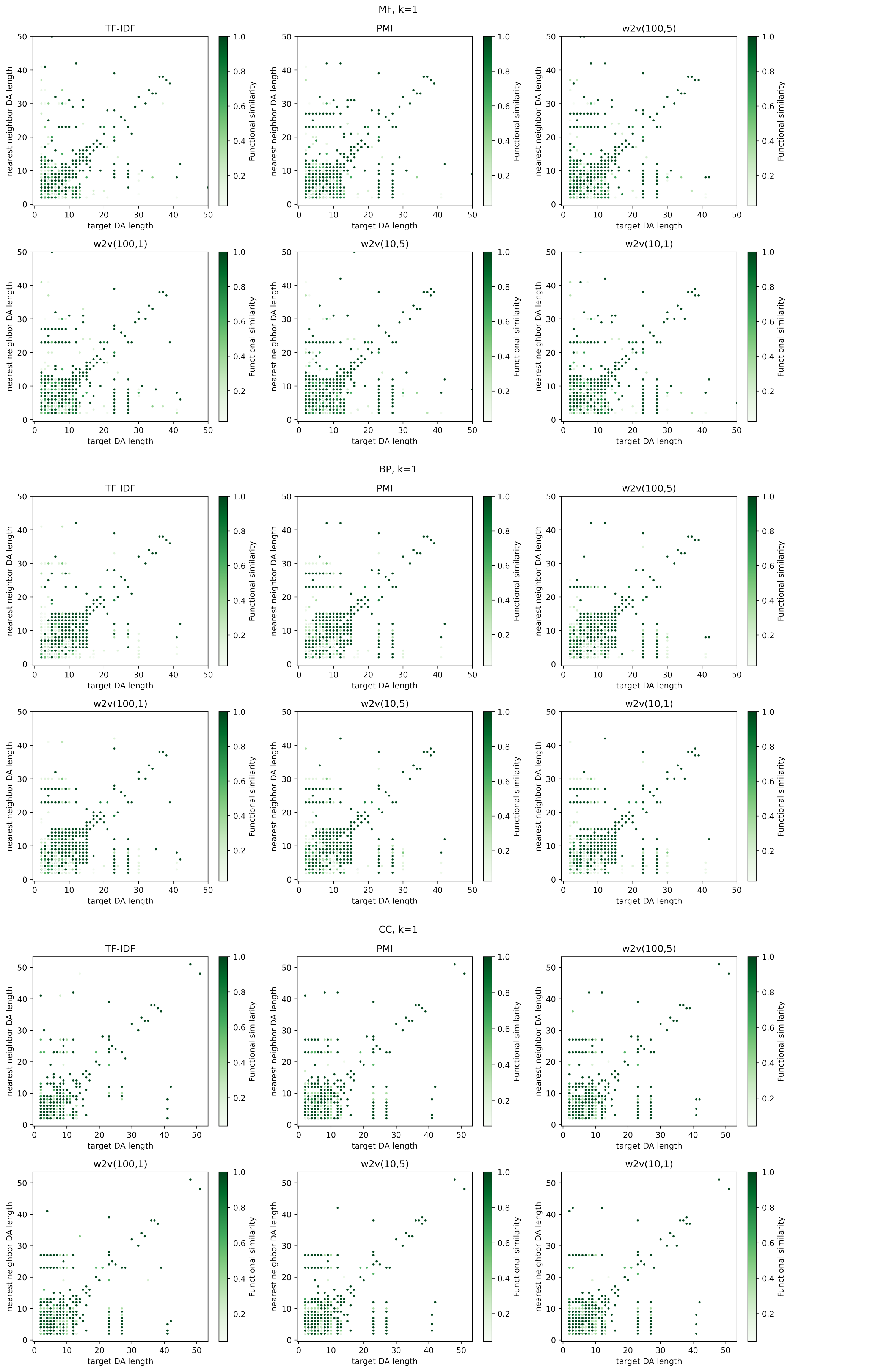

Fig. S4. Scatter plot of lengths of target domain architectures and domain-sharing nearest neighbors ($k = 1$). Functional similarity is color-coded.

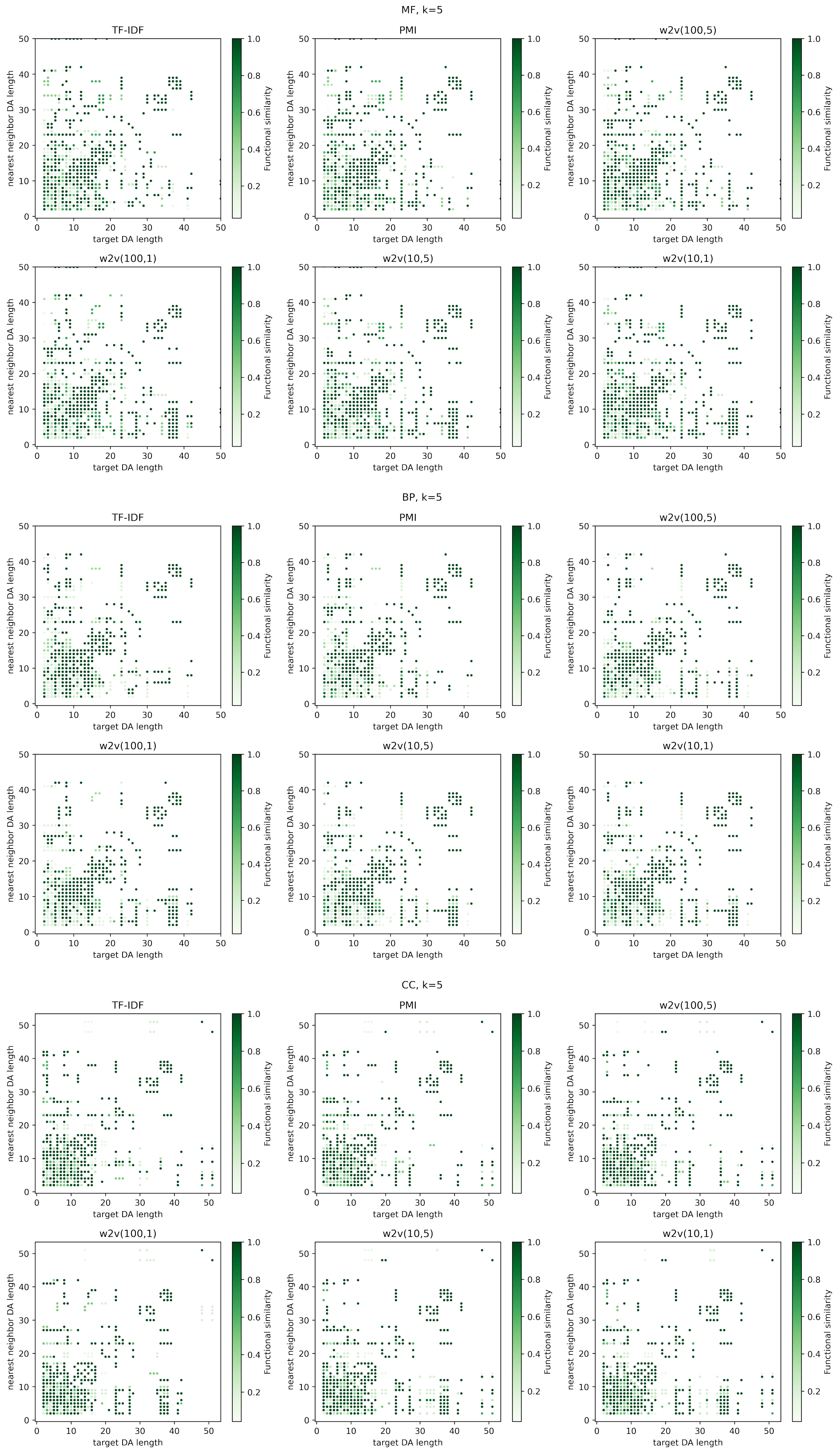

Fig. S5. Scatter plot of lengths of target domain architectures and domain-sharing nearest neighbors ($k = 5$). Functional similarity is color-coded.

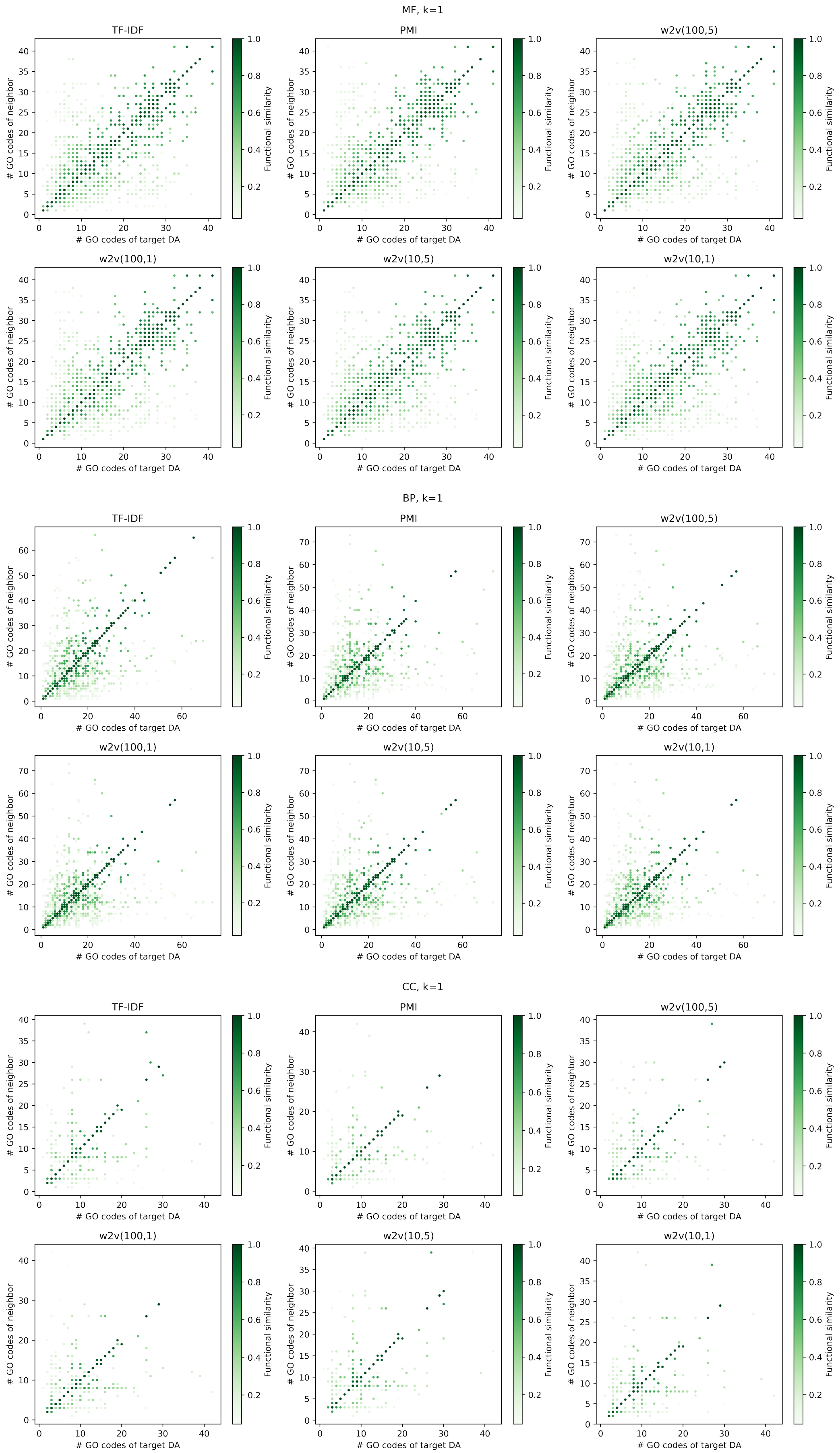

Fig. S6. Scatter plot of the number of GO codes of target domain architectures and domain-sharing nearest neighbors ($k = 1$). Functional similarity is color-coded.

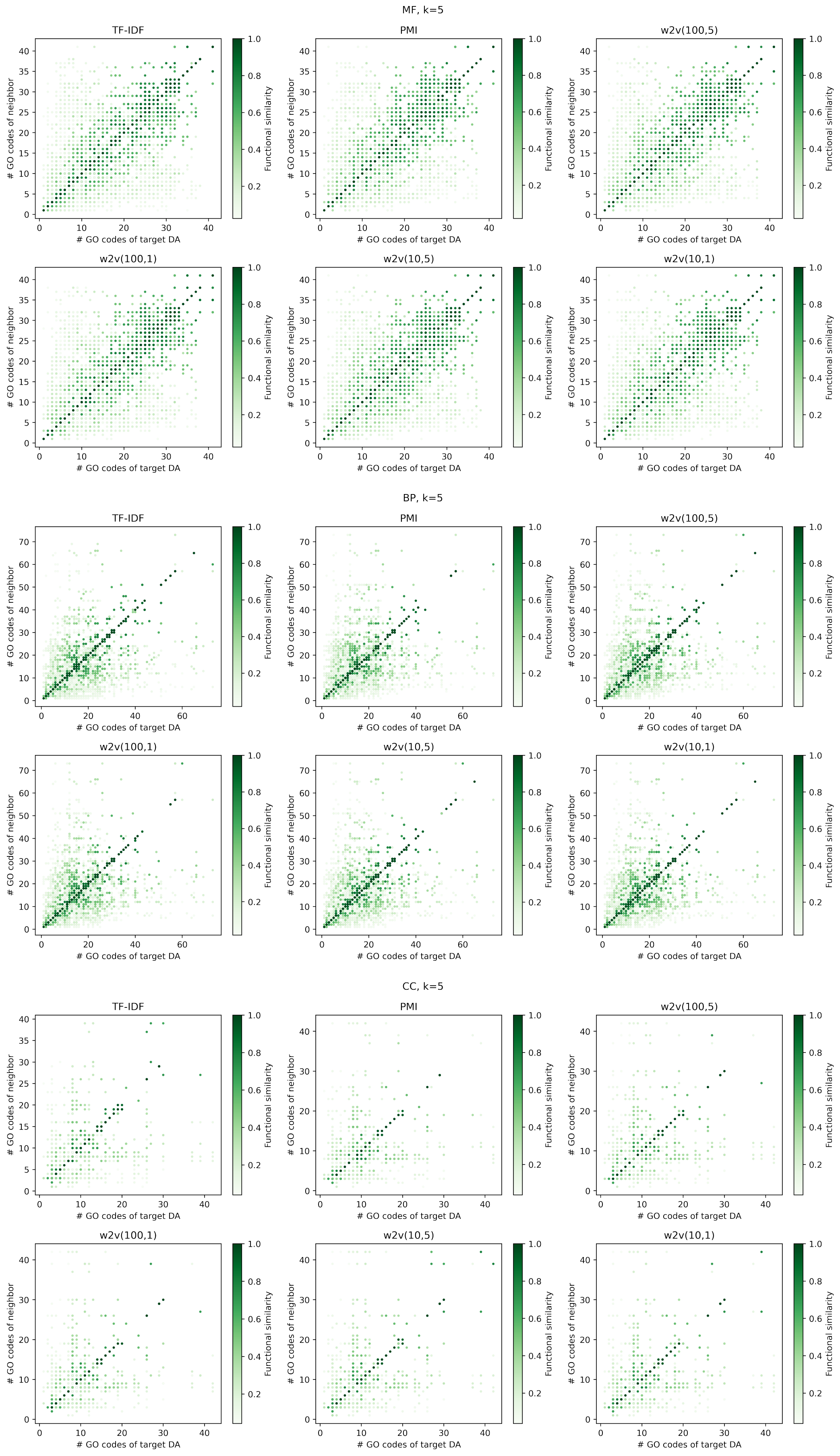

Fig. S7. Scatter plot of the number of GO codes of target domain architectures and domain-sharing nearest neighbors ($k = 5$). Functional similarity is color-coded.

Nearest neighbors that share no domains

Table S7. Average functional similarity between nearest neighbors that lack a common domain (Eqn. 9).

MF	$k = 1$	$k = 3$	$k = 5$	$k = 10$
TF-IDF	0.114	0.188	0.188	0.192
PMI	0.161	0.153	0.153	0.147
w2v(100,5)	0.178	0.194	0.215	0.199
w2v(100,1)	0.184	0.179	0.172	0.168
w2v(10,5)	0.169	0.191	0.203	0.205
w2v(10,1)	0.188	0.183	0.196	0.178
w2v(5,5)	0.214	0.209	0.207	0.188
w2v(5,1)	0.213	0.220	0.207	0.187

BP	$k = 1$	$k = 3$	$k = 5$	$k = 10$
TF-IDF	0.095	0.168	0.168	0.174
PMI	0.162	0.140	0.143	0.126
w2v(100,5)	0.187	0.173	0.179	0.174
w2v(100,1)	0.177	0.180	0.176	0.166
w2v(10,5)	0.174	0.180	0.182	0.170
w2v(10,1)	0.182	0.170	0.170	0.163
w2v(5,5)	0.186	0.182	0.181	0.180
w2v(5,1)	0.190	0.188	0.181	0.174

CC	$k = 1$	$k = 3$	$k = 5$	$k = 10$
TF-IDF	0.406	0.381	0.346	0.355
PMI	0.270	0.265	0.237	0.216
w2v(100,5)	0.305	0.307	0.314	0.311
w2v(100,1)	0.430	0.342	0.355	0.320
w2v(10,5)	0.369	0.334	0.331	0.301
w2v(10,1)	0.344	0.307	0.303	0.288
w2v(5,5)	0.365	0.350	0.341	0.290
w2v(5,1)	0.385	0.354	0.341	0.308

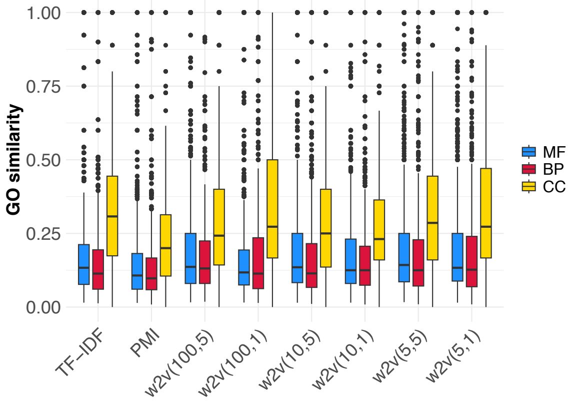

Fig. S8. Functional similarity between target domain architectures and the $k = 5$ nearest neighbor that lacks a common domain. GO terms associated with each target are compared to the union of GO terms from neighbors.

DA pairs that lack shared domains but have high functional similarity

The list of pairs of DAs that are identified as nearest neighbors ($k = 1$) that lack a shared domain, but have functional, similarity above 0.8, is provided as candidates.xlsx at `https://zenodo.org/records/15769961/preview/zenodo.zip?preview=1&include_deleted=0#tree_item5`

Comparison with domain embedding

Table S8. Accuracy of GO annotation transfer in the k-neighborhood for domains, following the protocol of Buchan and Jones [2020].

**Molecular Function**	$k = 1$			$k = 3$			$k = 5$			$k = 10$		
	precision	recall	MCC	precision	recall	MCC	precision	recall	MCC	precision	recall	MCC
TF-IDF	0.276	0.407	0.276	0.176	0.572	0.271	0.147	0.619	0.256	0.125	0.649	0.237
PMI	0.288	0.354	0.261	0.213	0.504	0.283	0.174	0.576	0.272	0.127	0.665	0.243
w2v(100,5)	0.313	0.302	0.259	0.208	0.483	0.270	0.169	0.571	0.264	0.122	0.659	0.234
w2v(100,1)	0.314	0.317	0.265	0.207	0.493	0.272	0.169	0.572	0.263	0.121	0.673	0.235
w2v(10,5)	0.313	0.310	0.266	0.210	0.497	0.278	0.169	0.582	0.267	0.120	0.674	0.235
w2v(10,1)	0.312	0.327	0.268	0.207	0.497	0.274	0.168	0.580	0.266	0.121	0.675	0.237
w2v(5,5)	0.318	0.311	0.267	0.207	0.488	0.272	0.170	0.573	0.265	0.125	0.675	0.241
w2v(5,1)	0.319	0.327	0.275	0.211	0.509	0.281	0.170	0.583	0.268	0.123	0.670	0.237

**Biological process**	$k = 1$			$k = 3$			$k = 5$			$k = 10$		
	precision	recall	MCC	precision	recall	MCC	precision	recall	MCC	precision	recall	MCC
TF-IDF	0.246	0.356	0.244	0.158	0.495	0.237	0.139	0.540	0.231	0.126	0.606	0.231
PMI	0.259	0.323	0.238	0.170	0.461	0.232	0.133	0.533	0.221	0.104	0.633	0.209
w2v(100,5)	0.278	0.275	0.225	0.160	0.438	0.218	0.125	0.523	0.208	0.088	0.635	0.187
w2v(100,1)	0.294	0.276	0.235	0.161	0.436	0.217	0.128	0.535	0.214	0.091	0.642	0.192
w2v(10,5)	0.275	0.285	0.230	0.163	0.454	0.226	0.125	0.529	0.210	0.091	0.644	0.193
w2v(10,1)	0.283	0.278	0.229	0.155	0.438	0.213	0.120	0.521	0.204	0.090	0.644	0.192
w2v(5,5)	0.287	0.293	0.239	0.162	0.448	0.224	0.129	0.533	0.216	0.092	0.641	0.193
w2v(5,1)	0.288	0.286	0.237	0.157	0.439	0.216	0.125	0.524	0.209	0.091	0.642	0.192

**Cellular component**	$k = 1$			$k = 3$			$k = 5$			$k = 10$		
	precision	recall	MCC	precision	recall	MCC	precision	recall	MCC	precision	recall	MCC
TF-IDF	0.397	0.564	0.394	0.223	0.737	0.342	0.184	0.779	0.320	0.162	0.799	0.299
PMI	0.413	0.558	0.412	0.272	0.696	0.374	0.196	0.756	0.324	0.141	0.802	0.275
w2v(100,5)	0.491	0.468	0.412	0.303	0.649	0.378	0.235	0.727	0.350	0.156	0.800	0.289
w2v(100,1)	0.487	0.455	0.402	0.285	0.644	0.365	0.222	0.715	0.337	0.151	0.796	0.285
w2v(10,5)	0.474	0.485	0.416	0.287	0.658	0.373	0.224	0.731	0.343	0.150	0.792	0.281
w2v(10,1)	0.467	0.483	0.406	0.282	0.665	0.370	0.216	0.729	0.334	0.151	0.791	0.281
w2v(5,5)	0.460	0.479	0.405	0.282	0.641	0.363	0.220	0.718	0.336	0.152	0.790	0.283
w2v(5,1)	0.448	0.468	0.385	0.287	0.655	0.367	0.225	0.728	0.341	0.156	0.796	0.288

Functional similarity in the k-neighborhood

Table S9. Mean functional similarity between nearest neighbors, $(S_F^O(\mathcal{N}_{O,k}(A), \mathcal{N}_{O,k}(A))$ averaged over all $A \in \mathcal{A}_O$), regardless of domain content sharing.

MF	$k=1$	$k=3$	$k=5$	$k=10$
TF-IDF	0.725	0.645	0.597	0.530
PMI	0.654	0.574	0.531	0.476
w2v(100,5)	0.690	0.611	0.569	0.511
w2v(100,1)	0.667	0.582	0.538	0.482
w2v(10,5)	0.681	0.610	0.569	0.513
w2v(10,1)	0.670	0.594	0.553	0.501
w2v(5,5)	0.589	0.519	0.486	0.443
w2v(5,1)	0.558	0.493	0.466	0.427

BP	$k=1$	$k=3$	$k=5$	$k=10$
TF-IDF	0.679	0.578	0.522	0.446
PMI	0.573	0.486	0.448	0.396
w2v(100,5)	0.616	0.528	0.487	0.430
w2v(100,1)	0.594	0.502	0.458	0.405
w2v(10,5)	0.617	0.538	0.493	0.434
w2v(10,1)	0.604	0.516	0.479	0.422
w2v(5,5)	0.523	0.461	0.430	0.388
w2v(5,1)	0.498	0.431	0.404	0.366

CC	$k=1$	$k=3$	$k=5$	$k=10$
TF-IDF	0.775	0.694	0.652	0.597
PMI	0.692	0.621	0.586	0.538
w2v(100,5)	0.739	0.656	0.623	0.576
w2v(100,1)	0.729	0.641	0.603	0.556
w2v(10,5)	0.739	0.664	0.629	0.580
w2v(10,1)	0.725	0.641	0.611	0.565
w2v(5,5)	0.684	0.611	0.583	0.544
w2v(5,1)	0.655	0.577	0.554	0.524

Table S10. Accuracy of GO annotation transfer in the k-neighborhood for multidomain architectures as the mean $S_F^O(A, \mathcal{N}_{O,k}(A))$ over all $A \in \mathcal{A}_O$, regardless of domain content sharing.

**Molecular Function**	$k = 1$			$k = 3$			$k = 5$			$k = 10$		
	precision	recall	MCC	precision	recall	MCC	precision	recall	MCC	precision	recall	MCC
TF-IDF	0.827	0.811	0.796	0.671	0.879	0.735	0.568	0.902	0.675	0.410	0.926	0.572
PMI	0.757	0.761	0.735	0.569	0.844	0.659	0.463	0.874	0.596	0.331	0.905	0.499
w2v(100,5)	0.787	0.789	0.766	0.615	0.861	0.697	0.511	0.884	0.635	0.374	0.910	0.541
w2v(100,1)	0.772	0.767	0.746	0.582	0.844	0.668	0.471	0.872	0.602	0.343	0.903	0.512
w2v(10,5)	0.783	0.778	0.758	0.619	0.858	0.698	0.517	0.882	0.638	0.385	0.908	0.548
w2v(10,1)	0.776	0.767	0.748	0.598	0.846	0.678	0.497	0.873	0.620	0.368	0.902	0.532
w2v(5,5)	0.705	0.704	0.677	0.529	0.809	0.618	0.441	0.846	0.571	0.325	0.888	0.493
w2v(5,1)	0.680	0.676	0.649	0.499	0.786	0.590	0.411	0.831	0.544	0.304	0.878	0.472

**Biological process**	$k = 1$			$k = 3$			$k = 5$			$k = 10$		
	precision	recall	MCC	precision	recall	MCC	precision	recall	MCC	precision	recall	MCC
TF-IDF	0.762	0.770	0.741	0.590	0.845	0.674	0.490	0.864	0.614	0.343	0.902	0.515
PMI	0.657	0.679	0.644	0.480	0.777	0.577	0.391	0.819	0.526	0.272	0.864	0.441
w2v(100,5)	0.709	0.710	0.687	0.532	0.802	0.622	0.437	0.836	0.568	0.309	0.874	0.479
w2v(100,1)	0.690	0.690	0.667	0.503	0.792	0.599	0.403	0.823	0.539	0.284	0.865	0.455
w2v(10,5)	0.710	0.708	0.686	0.544	0.805	0.630	0.446	0.836	0.574	0.316	0.873	0.484
w2v(10,1)	0.700	0.694	0.675	0.521	0.790	0.610	0.436	0.828	0.563	0.305	0.867	0.473
w2v(5,5)	0.627	0.628	0.602	0.471	0.753	0.563	0.392	0.800	0.523	0.278	0.853	0.447
w2v(5,1)	0.603	0.612	0.582	0.436	0.734	0.533	0.360	0.782	0.493	0.257	0.840	0.426

**Cellular component**	$k = 1$			$k = 3$			$k = 5$			$k = 10$		
	precision	recall	MCC	precision	recall	MCC	precision	recall	MCC	precision	recall	MCC
TF-IDF	0.842	0.851	0.829	0.696	0.909	0.764	0.596	0.925	0.702	0.464	0.944	0.609
PMI	0.781	0.791	0.762	0.622	0.875	0.702	0.518	0.898	0.636	0.386	0.929	0.542
w2v(100,5)	0.828	0.819	0.803	0.661	0.881	0.729	0.569	0.901	0.675	0.434	0.928	0.585
w2v(100,1)	0.824	0.806	0.794	0.649	0.879	0.721	0.554	0.898	0.664	0.416	0.922	0.570
w2v(10,5)	0.829	0.815	0.802	0.684	0.878	0.742	0.592	0.900	0.690	0.445	0.927	0.592
w2v(10,1)	0.812	0.807	0.789	0.658	0.874	0.725	0.568	0.900	0.674	0.431	0.928	0.582
w2v(5,5)	0.783	0.777	0.757	0.625	0.863	0.698	0.530	0.890	0.644	0.393	0.923	0.551
w2v(5,1)	0.766	0.752	0.733	0.578	0.846	0.661	0.497	0.884	0.620	0.380	0.916	0.540
